# Nanoparticle suspensions from carbon-rich fluid make high-grade gold deposits

**DOI:** 10.1038/s41467-022-31447-5

**Published:** 2022-07-01

**Authors:** Laura Petrella, Nicolas Thébaud, Denis Fougerouse, Brian Tattitch, Laure Martin, Stephen Turner, Alexandra Suvorova, Sarah Gain

**Affiliations:** 1 Centre for Exploration Targeting, University of Western Australia, Crawley, Australia; 2 School of Earth and Planetary Sciences, Curtin University, Bentley, Australia; 3 Centre for Microscopy Characterisation and Analysis, The University of Western Australia, Perth, WA Australia; 4 Newmont Corporation, Welshpool, WA Australia; 5 Geological Survey of Western Australia, Perth, WA Australia

**Keywords:** Geochemistry, Economic geology, Geology

## Abstract

Economic gold deposits result from a 100- to 10,000-fold enrichment in gold relative to crustal background. In hydrothermal systems, this enrichment is achieved through the transport and accumulation of metals via deeply sourced fluids to a site of deposition. However, the generally low metal solubility of Au in aqueous solutions in orogenic systems requires additional processes in order to explain high-grade gold formation. Reports of Au nanoparticles in high-grade gold veins infer that their formation is linked to mineralisation. However, processes leading to nanoparticle nucleation and deposition remain poorly understood. Here we show that formation of metal nanoparticles (Au, AuAg, Cu, Ag_2_O) is one of the essential contributors to efficient and focused gold deposition. We report systematic and previously unrecognized metal nanoparticles preserved in amorphous silica and/or carbonic phases in five high-grade deposits. The association of metal, silica and carbonic phases helps to constrain the multiple reactive processes involved in Au, Cu and Ag metallogenesis and formation of high-grade gold mineralisation.

## Abstract (web summary)

The authors present novel observations providing insights into the formation of extraordinary gold-rich veins. We discovered metal nanoparticles associated with amorphous silica and carbon indicating their essential contribution to efficient gold deposition.

## Introduction

**Fig. 1:**
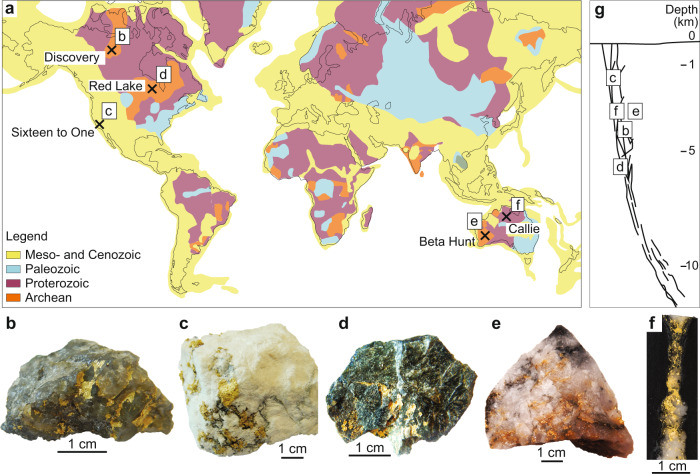
Sample location and description. **a** Location of the deposits from which samples were collected for this study; the deposit locations are shown on a world map indicating the thermo-tectonic age of the country rocks, modified from USGS thermo-tectonic data^50^. **b** to **f** show photographs of the gold-rich quartz vein specimen used in this study: **b** Discovery; **c** Sixteen to One; **d** Red Lake; **e** Beta Hunt; **f** Callie. **g** Schematic cross-section showing the estimated depth of formation of the deposits studied.

Gold has played a critical role in the rise and collapse of human civilizations from the fifth millennium BC in Egypt through to the modern era^1^. This rare transition metal, with unique physical properties, is primarily extracted from mineral deposits. Mineral deposits are formed through the circulation of gold-bearing, aqueous solutions in the Earth’s interior^2^. Orogenic-type gold deposits, which account for over 75% of the world’s gold production^3^, characteristically contain high-grade gold in quartz veins. Orogenic gold deposits are typically formed within metamorphic belts in the upper continental crust, through a network of veins and faults, which focus deeply-sourced aqueous solutions towards shallower levels in the crust^4,5^. In most orogenic-type gold deposits, the mineralising aqueous fluids have characteristic temperatures of ~250 to 450 °C, pressures from 500 to 1500 bars, low salinities (generally ≤ 3 wt% NaCl equivalent), high CO_2_ and H_2_S content and a near neutral pH^6^. From their deeply sourced reservoir(s) to the deposition site, gold is transported in hydrothermal solutions in the form of dissolved complexes^7^ that achieve maximum solubilities of hundreds of ppb^7,8^. Most models propose that the repeated percolation of large amounts of fluid through faults and veins is necessary for the formation of extremely high-grade gold (~10,000 ppm Au) veins^9,10^. Some types of high-grade gold veins (e.g., Figure 1b to f) are narrow (few mm to cm) with a non-laminated texture indicative of only one fluid percolation event. The veins are associated with a narrow alteration footprint and growth zoning was not detected in the vein-filling minerals^11^. Collectively, these observations do not support repeated or large fluid-fluxing events^10,12,13^. Instead, the textural characteristics and alteration footprint documented in these high grade gold veins point towards a single injection of a gold bearing solution, an interpretation which is difficult to reconcile with the generally low gold solubilities assumed for orogenic hydrothermal solutions^8^.

To address this long-standing dilemma, recent work has proposed that gold might be transported as a nanoparticle (NP) suspension^14–20^. Gold NP suspensions can concentrate up to ~5,000 times more gold in fluid than when gold is a dissolved species^21^, thus suspensions offer a viable additional mechanism for metal transport in aqueous fluids. However, direct evidence for Au NPs in orogenic gold systems is limited^22,23^.

Our study of natural samples from high-grade gold mines presents new data that allow us to peer into the processes of nucleation, stabilisation and deposition of Au NPs associated with the formation of high-grade orogenic gold deposits. Exceptionally high-grade, gold-rich quartz vein samples were collected from five gold deposits characterised by the presence of coarse visible gold that formed at a range of crustal depths, from ~1.5 km to >5 km below the paleo-surface^24–28^. The deposits formed in different host lithologies and have mineralisation ages ranging from the Archean to the Cretaceous^11,29–33^ (Fig. 1).

## Results

The veins selected for this study represent exceptionally high-grade Au-rich samples that contain coarse gold grains. They do not display laminated textures which suggests that they formed during one opening event^10,12^. The samples studied are representative of veins that are very nuggety and found within very high-grade orogenic orebodies. The main vein filling minerals is quartz, in which growth zoning was not detected under scanning electron microscope-cathodoluminescence (SEM-CL) imaging. Few veins contain pyrite, in which again no growth zoning was detected. The lack of zoning in quartz and pyrite argues against repeated or large fluid fluxing events in these mineralizing systems.

One-inch epoxy mounts with coarse gold was prepared from each vein sample. One to four ultra-thin Transmission Electron Microscopy (TEM) foils (10 × 2 µm and ~100 nm in thickness) were extracted from gold grains in each of these mounts using Focused Ion Beam Scanning Electron Microscopy (FIB-SEM). The foils were extracted from areas of interest containing inclusions. The gold grains were carefully selected based on the presence of intergrowth texture with quartz in the vein. Grains located in secondary, cross-cutting features within the vein were not considered for this study, because these might represent a remobilised or late-mineralisation phase. The foils were then investigated by TEM.

### Micro-inclusions in gold and metal nanoparticles

**Fig. 2:**
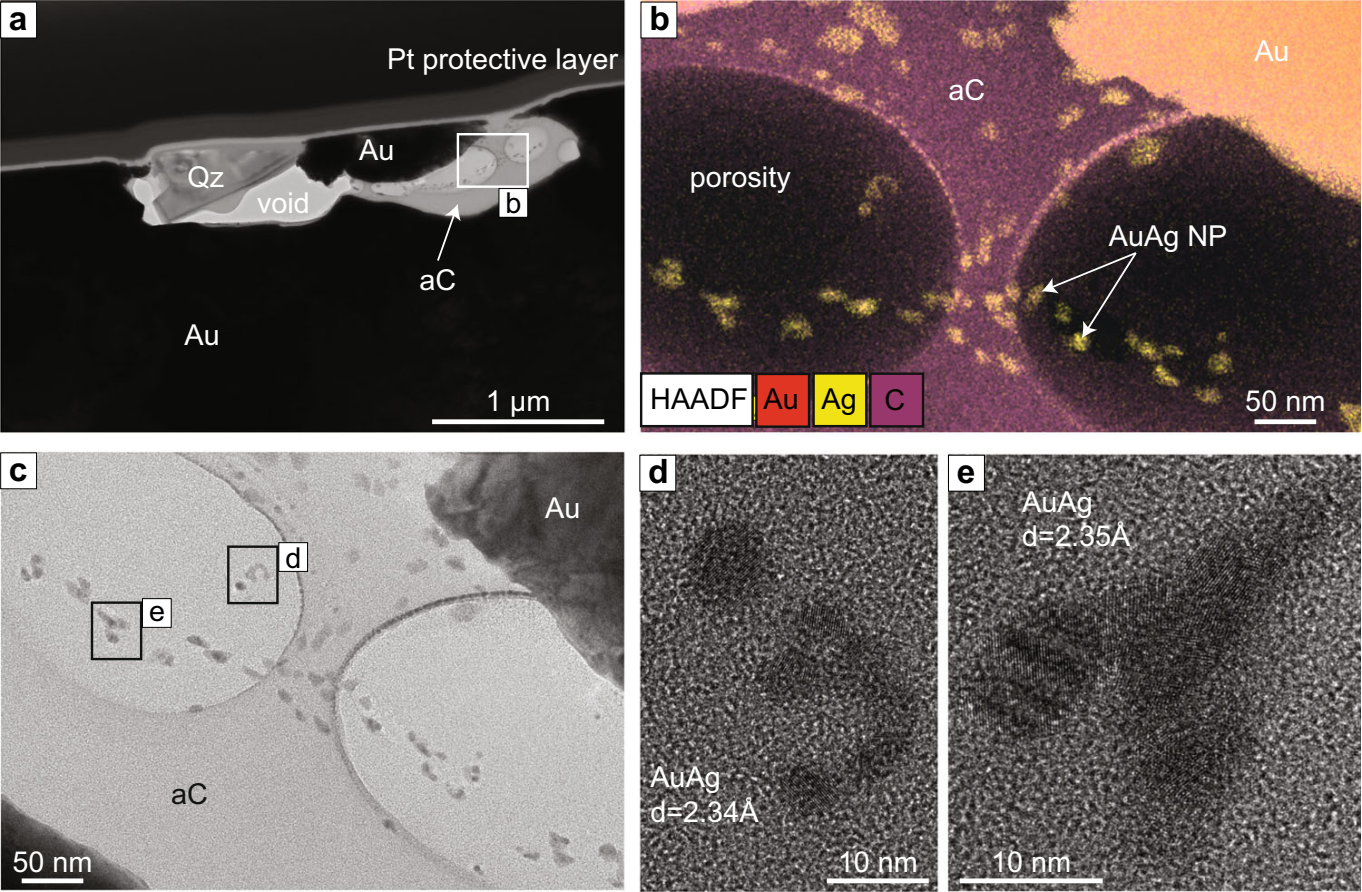
Red Lake electrum nanoparticles in amorphous carbon. **a** High-Annular Dark Field Scanning Transmission Electron Microscopy (HAADF-STEM) image of the Red Lake foil showing elongated inclusion at the surface of the foil, mainly composed of quartz (Qz) and an amorphous carbon phase (aC); the rest of the foil comprises coarse gold; the top of the foil is covered by a protective platinum (Pt) layer. **b** Energy-dispersive X-ray spectroscopy (EDS) elemental map overlaying a HAADF-STEM image from the middle of the inclusion, indicated by white rectangle in Fig. 2a; electrum nanoparticles (NPs) are encapsulated in a carbon-rich phase. **c** TEM image of the area marked by white rectangle in Fig. 2a; the inclusion is composed of an amorphous carbon phase (light grey) containing sub-rounded NPs (darker grey). Two large, “bubble”-like features correspond to cavities within the amorphous phase. **d**, **e** High Resolution TEM (HRTEM) image of areas marked by black rectangles in Fig. 2c showing electrum NPs with sizes varying from 4 to 10 nm. The FFT collected from the largest NP confirms a d-spacing of 2.34 Å, corresponding to Ag or Au or Ag-Au alloy.

One foil was extracted from a gold grain in the Red Lake sample which contains a single ~3 µm elongated inclusion located close to the surface of the foil (Fig. 2a). TEM analyses show that the inclusion consists of three components: quartz, metal NPs, and an amorphous carbonic phase. The elemental distribution of the phases was identified by Scanning Transmission Electron Microscopy Energy-dispersive X-ray spectroscopy (STEM-EDS) mapping (Fig. 2b). The absence of crystallinity in the silica and carbonic phase was confirmed by Fast Fourier Transform (FFT) diffractograms acquired from both phases. The amorphous carbonic phase preserves bubble-like textures; these could be due to primary porosity from fluid inclusions released during the foil preparation or they could be an artefact caused by inhomogeneous milling during foil preparation. The latter could be due to the softer nature of amorphous carbon compared to the coarse gold. The amorphous carbonic phase encapsulates numerous rounded NPs that are locally aggregated (Fig. 2c–e). The size of the NPs varies from 2 nm (for single NP) to 20 nm (for the nanoparticle aggregates) (Fig. 2e). The d-spacing of the nanoparticles varies between 2.34 Å and 2.35 Å, indicating the presence of metallic Au (111), Ag (111) or AuAg (111). A quantitative composition obtained by EDS on one NP indicates ~54 at% Au, ~41 at% Ag and ~5 at% O (Supplementary Information, Table 3), thus is classified as electrum. The presence of O most likely derives from a beam overlap of the NP examined with the surrounding amorphous carbonic phase that contains elevated O.

**Fig. 3:**
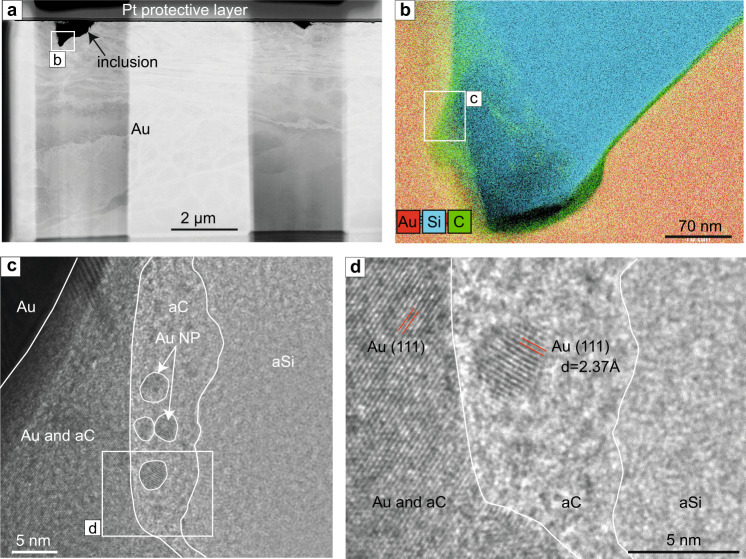
Beta Hunt nanoparticles in amorphous carbon and silica. **a** High-Annular Dark Field Scanning Transmission Electron Microscopy (HAADF-STEM) image of the entire Beta Hunt foil showing a large inclusion on the left-hand side and a smaller inclusion on the right-hand side of the foil. **b** Energy-dispersive X-ray spectroscopy (EDS) elemental map of the part of the largest inclusion (indicated by a white rectangle in Fig. 3a) showing amorphous silica in the centre of the inclusion and a thin carbon layer at the interface with gold. **c** Transmission Electron Microscopy (TEM) image of the area within the white rectangle in Fig. 3b. The image shows amorphous silica (aSi) in the right-side of the image, amorphous carbon (aC) concentrated along the interface between the amorphous silica and the host gold, numerous rounded Au or Ag NPs (Au NP) are encapsulated in the amorphous carbon phase. **d** High Resolution TEM image of the area indicated by a white rectangle in Fig. 3c. showing 5 nm Au (or Ag) NP in amorphous carbon. A d-spacing of 2.37 Å was obtained for the metal NP using FFT analysis and was matched with Au, Ag, or Au-Ag alloy crystal structures.

One foil extracted from a gold grain from the Beta Hunt sample contains one inclusion that is c. 1.5 µm in diameter (Fig. 3a) and is composed of two phases: a silica phase that occupies the middle part of the inclusion and a thinner, homogeneous carbonic phase located along the edge of the inclusion (Fig. 3b). A High-Resolution Transmitted Electron Microscopy (HRTEM) image does not show any crystalline features within either phase, and the FFT diffractograms from both areas confirm that the phases are amorphous. As the TEM image is a projection through the entire thickness of the sample, in some areas the various phases might overlap (Fig. 3c). Rounded gold or silver NP were observed in the amorphous carbonic phase (Fig. 3c, b). The NPs are small (3 to 5 nm) and concentrated within a thin layer of the amorphous carbonic phase near the contact with the gold crystal. A d-spacing of 2.37 Å was measured for the NPs, with the lattice orientation of (111) indicative of gold or silver. Due to the small size of the NPs, the EDS signal was not sufficient to determine whether they were composed of Au, Ag or Au-Ag. Other Au NPs were observed in the amorphous silica phase within the upper part of the inclusion (Supplementary Information, Fig. 7).

**Fig. 4:**
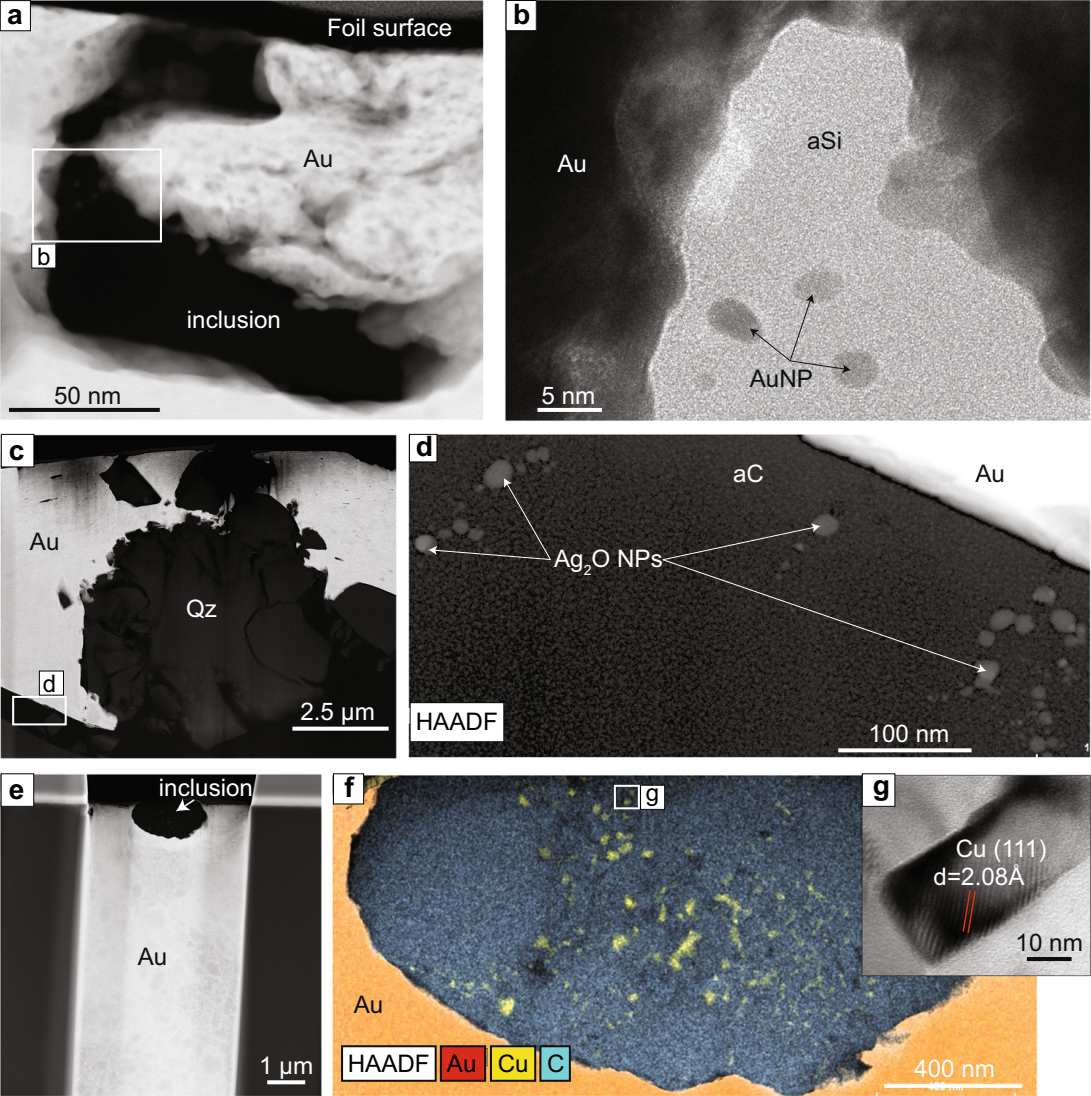
Gold, silver oxide and copper nanoparticles in amorphous carbon and silica. **a** High-Annular Dark Field Scanning Transmission Electron Microscopy (HAADF-STEM) image of the inclusion in Discovery sample foil 1. **b** TEM image of the area indicated by a white rectangle in Fig. 4a that shows the amorphous silica (aSi) phase filling the inclusion and round-shape Au nanoparticles (Au NP). **c** HAADF-STEM image of Discovery foil 2: the light grey crystalline phase corresponds to coarse gold (Au), the dark grey phase corresponds to quartz (Qz) and the black phase within the foil is an amorphous carbon phase (aC). **d** HAADF image of the area indicated by a white rectangle in Fig. 4c showing round-shape Ag_2_O NPs within the amorphous carbon phase. **e** HAADF-STEM image of the Sixteen to One foil that shows the inclusion analysed located at the surface of the foil. **f** Energy-dispersive X-ray spectroscopy (EDS) elemental map overlaying a HAADF-STEM image of the Sixteen to One inclusion showing a composition of micro-crystalline carbon with interstitial Cu NPs. **g** HRTEM image of the area indicated by a rectangle in Fig. 4f that shows a 50 nm Cu NP.

Two foils were extracted in gold grains from the Discovery sample. Foil 1 (Fig. 4a) contains an elongated inclusion of ~1.5 µm × 0.5 µm in size. The TEM image of the inclusion shows that an amorphous silica phase fills the inclusion and NPs are encapsulated within this phase (Fig. 4b). The NPs form sub-rounded crystals with diameters that vary from 5 to 8 nm. The EDS map acquired from the area with the inclusion indicates that the amorphous phase is composed of silica and the four NPs are composed of Au (Supplementary Information, Figure 8). The absence of crystallinity in the silica phase is confirmed by a FFT diffractogram. The d-spacing of 2.34 Å (111) obtained from FFT diffractograms on the NPs indicates that the mineral species is Au.

The second foil (foil 2) contains quartz fragments and coarse gold that are surrounded by an interstitial amorphous carbonic phase (Fig. 4c). The elemental distribution of each phase in foil 2 was analysed by EDS elemental maps and the crystallinity state was analysed by HRTEM and FFT diffractograms (Supplementary Information, Figure 9). Within the lower part of foil 2, approximately 20 NPs were identified within the amorphous carbonic phase (Fig. 4d). EDS spectra acquired from the NPs indicate Ag and O and FFT diffractograms indicate d-spacings of 1.94 Å (112), 2.78 Å (111) and 3.20 Å (101), indicative of a Ag_2_O cubic lattice. The diameter of the Ag_2_O NPs varies from 8 to 15 nm.

The observations reported from the Callie TEM foil have been extended from Petrella, et al.^22^ who reported Au NPs preserved in amorphous silica in one domain of an inclusion in coarse gold from a quartz vein. In this study we acquired an EDS elemental map of the same entire inclusion rather than only the area that contains the Au NPs. The whole-inclusion elemental map acquired in this study (Supplementary Information, Fig. 10) revealed that only one side of the inclusion is composed of amorphous silica whereas the rest of the inclusion is composed of an amorphous carbonic phase.

One foil (Fig. 4e) was extracted from the Sixteen to One sample, in a coarse gold grain bearing numerous carbon-rich inclusions <3 µm in size. The foil contains a 2 µm sub-rounded inclusion, mainly composed of crystalline micro-grains of carbon which vary in size from approximately 20 nm to 100 nm. Within the inclusion, there are more than 20 Cu NPs interstitial to the crystalline carbon (Fig. 4f). The composition and structure of the NPs was confirmed using EDS and FFT diffractograms that show a d-spacing of 2.08 Å, corresponding to Cu (Fig. 4g). The Cu NPs are comparatively large with sizes varying from 10 nm to 100 nm and display sub-angular crystal shapes in contrast with the sub-rounded Au, AuAg and Ag_2_O NPs.

Quantitative chemical composition of the amorphous phases associated with the NPs was acquired in all samples (composition in Supplementary Information, Table 3). An amorphous silica phase was identified in inclusion in gold grain from the Beta Hunt, the Discovery and the Callie samples. Measured amorphous silica compositions in all the sample include Si (varies from ~38 at% to ~56 at%), O (varies from ~44 at% to ~62 at%) and minor or trace amount of Au (<1.5 at%). An amorphous carbonic phase was identified in inclusion in gold grain from Beta Hunt, Discovery, Red Lake and Callie samples. The amorphous carbonic phase compositions include C (varies from ~70 at% to ~93 at%), O (varies from ~4 at% to ~19 at%) and N (varies from ~1 at% to ~3 at%, with minor or trace amount of Au (<1 at%).

## Discussion

In this study we report the presence of Au, Cu, AuAg and Ag_2_O NPs preserved in micro-inclusions in gold grains. The inclusions are filled with amorphous silica and/or an amorphous carbonic phase (containing C, O, N), or micro-crystalline carbon (in one sample: Sixteen to One). The size of the metal NPs varies from 1 to 100 nm with a majority being under 10 nm. The occurrence of metal NPs in all five investigated high-grade orogenic gold deposits demonstrates for the first time that: (1) the close association between metal NPs and high-grade orogenic gold mineralisation; (2) Au is not the only metal that occurs as NP but AgAu, Ag_2_O and Cu NPs can also form during metallogenic processes in high-grade orogenic gold systems; (3) There is an intimate association between metal NPs and amorphous silica and/or carbon-bearing phases in these systems. Such associations suggest a link between these phases and the metallogenic process leading to the formation of high-grade gold deposits. Below, we investigate how amorphous silica and carbon-bearing phases are associated with the nucleation, stabilisation and focused deposition of metal NPs within high-grade gold mineral systems.

A crucial outcome of this study is the novel documentation of the systematic association of metal NPs with carbonic phases. The amorphous carbonic phase observed in all but one deposits consists of C, O and N which are the same elements found in carbon-rich fluid inclusions common in quartz associated with gold in orogenic deposits^34–36^. The deposits investigated are hosted in a variety of rock types, some of which have carbon-poor compositions (e.g., basalt; host rock description in supplementary material), thus it is unlikely that the carbon was sourced from the host-rock. Fluid inclusion studies in quartz associated with orogenic gold indicate fluid compositions dominated by CO_2_ with minor CH_4_ and N_2_^36^. At the Callie deposit, fluid inclusions are composed of 50 to 100 mol% CO_2_, CH_4_ and minor N_2_^28^, whereas at Red Lake the fluid inclusions are dominated by CO_2_ with minor CH_4_, N_2_ and trace H_2_S^36^. The similarity between the composition of the amorphous carbonic phases in this study and that measured in fluid inclusions suggests that carbon-bearing phases are precipitated directly as an amorphous phase from the hydrothermal solution within the vein.

**Fig. 5:**
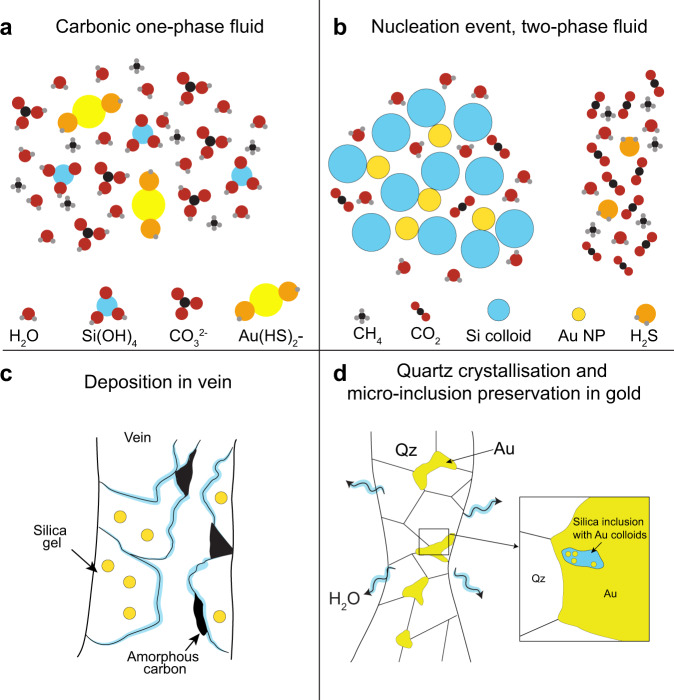
Proposed model for the formation of high-grade gold veins in orogenic system. **a** Simplified estimated composition of a one phase aqueous carbonic fluid initially present in the orogenic hydrothermal system prior to reaching site of deposition. The fluid contains dissolved metal species and acid silicic. **b** Simplified estimated composition of a two-phase fluid formed by flash vaporisation of the carbonic fluid in **a**. The phase separation leads to the nucleation of silica and metals NPs. **c** Schematic representation of the deposition of silica gel containing metal NPs and the precipitation of amorphous carbon in the vein. **d** Schematic representation of the crystallisation of quartz and coarse gold in a vein from amorphous silica bearing Au NPs modified after^22^. The diagram also illustrates how NPs-bearing amorphous silica may have been trapped as inclusions in gold grain.

Previous studies on the nature and source of hydrothermal solutions in orogenic systems^2,37^ assume that CO_2_ originates from the fluid source that produced a H_2_O-CO_2_ solution. The solution also carries dissolved species including, but not limited to, silicic acid and dissolved metals^16^. The Au concentration of these solutions may differ as Au can be complexed by various ligands, whose concentrations in these solutions vary depending on the thermo-chemical conditions and bulk composition of the fluid^7,38^ (Fig. 5a). Reduced sulfur is predicted to be the dominant ligand for Au in orogenic solutions^7,38,39^. The relative abundance of CO_2_ in the solutions associated with orogenic gold mineralisation is also commonly recorded in fluid inclusion compositions^34–36^. During Au transport in a H_2_O-CO_2_ fluid in a mid-crustal environment (e.g., 300–450 °C and 1,000+ bars), elevated concentrations of CO_2_ in the hydrothermal solution will buffer the fluid at a pH just below neutral (pH~6;^39^) resulting in a modest increase of the Au-HS complex stability^40^, thus increasing the concentration of dissolved Au in the solution, as described by Phillips and Evans^39^. The increase in the stability of hydrothermal Au complexes is paired with an increased capacity for CO_2_-rich fluids to resist external pH control by fluid-rock interactions^39^. In fact, the addition of significant Na and K through some fluid-rock interaction at high temperatures increases the HCO_3_ in CO_2_-rich fluids but results in a higher, i.e., moderated, pH (pH > 6)^39^. As a result, source fluids very rich in CO_2_ and H_2_S with cations such as K^+^ should maximize Au solubility due to a moderate pH between 6 and 8. In this mineral system, abundant HS^−^ will enhance the fluid’s ability to complex Au.

As CO_2_-rich fluids decompress and cool towards 200 °C, H_2_CO_3_ in the fluid becomes more acidic, buffering the pH towards values closer to pH~4. This change in the buffering properties of CO_2_ has been proposed to be connected to sharp decreases in Au solubility, especially when coupled with decreasing temperature^39^ and phase separation in CO_2_-rich fluids (see the section below). We argue that very high CO_2_ concentrations both significantly increase the initial gold available for mineralisation and subsequently cause focused Au deposition as fluids cool and decompress, resulting in the anomalous Au grades in the deposits studied.

We observed an intimate relationship between metal NPs and amorphous silica in most deposits studied. The amorphous silica phase is widely interpreted to be derived from the deposition of silica colloids as silica gel in the ore-bearing veins^15,16,22,41^. These silica colloids are a stable suspension of discrete particles (or sols) of amorphous silica in the H_2_O-CO_2_ fluid; when the silica particles start to coagulate they transition towards forming a hydrous precipitate in the form of a viscous gel^42^.

Our observations indicate that nucleation of metal NPs and colloidal silica is commonplace as they occur together regardless of the age, host rock composition or emplacement depth of the orogenic hydrothermal mineral system. As both colloidal silica and metal NPs occur together, we suggest that a single set of processes is responsible for the nucleation of both phases and that their formation occurred nearly simultaneously. The nucleation of metal NPs result from the supersaturation of the solution in the element(s) of interest^10,43^; this is linked to reduced solubility due to cooling and decompression of the fluid. We propose that the high-grade Au ore zones examined here formed as Au-enriched H_2_O-CO_2_ fluids underwent a series of highly localized changes in chemical and physical properties (Fig. 5b). These, in sequence, are: 1^st^) sharp decreases in Au solubility due to increasingly acidity due to the presence of dissolved CO_2_ (dissociation of H_2_CO_3_) and fluid cooling; 2^nd^) further destabilization of Au-HS complexes and formation of colloidal silica due to decompression and cooling; 3^rd^) a flash vaporisation event (via a process such as fault valve system^43^) inducing rapid precipitation of silica (formation of amorphous silica), formation of amorphous carbon, and precipitation/accumulation of native Au.

Metal NPs reported in this study can be as small as 1 nm and a majority are <10 nm in diameter indicating that some metal NPs did not grow to larger particle sizes by coalescence of neutral Au in solution or overgrowth of Au on existing particles due to short-lived Ostwald Ripening processes^44^. We propose that continued aggregation of metal NPs to form even larger gold grains may have been locally delayed due to adsorption of the metal NPs onto colloidal silica^21,45^ forming prior to or at the same time as the NPs. This adsorption may have limited substantial deposition of the metal in veins immediately after the supersaturation event.

The mineralisation event corresponds to the physical aggregation and deposition of the metal NPs and soluble gold in veins at the site of mineralisation. In our study, we observe a spatial association between metal NPs, amorphous silica and carbonic phases (under the form of amorphous carbon and micro-crystalline carbon) suggesting that the processes that led to their deposition are the same or at least significantly linked (Fig. 5c).

The amorphous silica preserved in the inclusions studied appears to be the remnant of a solidified silica gel that was deposited in the vein^16,22,41^ during mineralisation. These silica gels must have been derived from the coagulation of a colloidal silica suspension^42^, as the gel is too viscous to be significantly transported as a hydrothermal suspension. The formation of silica colloids is most likely the result of flash vaporisation (rapid decompression) of the solution^17^. We postulate that the same flash vaporisation event is responsible for the nearly coeval nucleation, aggregation (in the form of a gel) and deposition in the vein. Later, the gel solidified in the form of amorphous silica. Our observations suggest that this silica gel was deposited in the vein along with the metal NPs by a confluence of processes, namely, decreasing Au-HS solubility due to decompression, changes to fluid chemistry (as CO_2_ and H_2_S are lost to the escaping vapour), and overall decompression and cooling of the fluids. These processes require relatively sudden changes in fluid conditions, which is consistent with the rapid nucleation required to produce both Au NPs (and other metals NPs) as well as silica colloids from the fluid.

The amorphous carbon-bearing phase observed in our study appears to have resulted from the reduction or oxidation of CO_2_ or CH_4_, resepectively, present in the fluid and their precipitation as an amorphous phase. The presence of Ag_2_O NPs shows that substantial oxidation of metallic Ag occurred at this time and suggests that amorphous carbon may be linked to oxidation-reduction reactions associated with the mineralization process. If these oxidation and/or reduction reactions were efficient, they may have strongly affected the ƒH_2_S, contributing to Au precipitation. The elements present in the amorphous carbonic phases have comparable relative abundances (C ~70 at% to ~93 at%, O ~4 at% to ~19 at% and N from ~1 at% to ~3 at%) in each deposit, pointing towards a generalised process or processes responsible for the deposition of carbon. Based on our observations, linking the presence of amorphous carbon with silica and metals NP, suggests that the deposition of amorphous carbon is likely to have occurred simultaneously with the deposition of silica gel and by the same process of fluid flash vaporisation.

Over the mineralisation time interval, the removal of water resulted in the amorphous silica present in the vein crystallising into quartz^22^, which expelled gold NPs from their crystalline structure, and subsequently, amalgamated onto any existing gold grains in the quartz veins^16,22^. During gold crystallisation, we believe that some of the amorphous phases and the associated metal NPs initially present in the vein were trapped as inclusions in the coarse gold grains (Fig. 5d). The gold encapsulating the inclusions inhibited the release of water which prevented the crystallisation of amorphous silica into quartz^22,46^. The amorphous texture of the carbon-rich phase was also preserved except in one case (Sixteen to One sample) where it is interpreted to have recrystallised from amorphous carbon into micro grained carbon-bearing minerals.

The veins selected for this study are attributed to a single vein-opening event^10,12,43^. Given the limited solubility of gold in orogenic hydrothermal solutions, the formation of a high-grade gold vein in a single mineralising event requires the percolation of a solution pre-concentrated in Au in order to reach an economic grade^8^. The question remains as to the origin of this pre-concentration.

In an H_2_O-CO_2 _± NaCl ± KCl ± H_2_S fluid at 500–1500 bars, the maximum CO_2_ solubility in the H_2_O-rich phase will occur at ~350 to 450 °C^47^, similar to the conditions of fluid-generation estimated in the systems examined here. The high H_2_CO_3_ pH buffering capacity of CO_2_-rich fluids and increased stability of Au-HS complexes will increase the Au scavenging and transport potential of this fluid compared to CO_2_-poor fluids^38^. During fluid ascend from its source, drops in pressure will lead to a cooling of the system and would also result in fluid immiscibility over a substantial P-T range in the host hydrothermal solution of H_2_O-CO_2_-NaCl-H_2_S. This cooling will drive pH towards acidic values that supress pyrite formation compared to more rock-buffered near-neutral values^39^. The lack of pyrite formation will help to prevent “smearing” gold deposition throughout sulfurized fluid flow zones which are common in lower grade deposits. We speculate the efficient and focused gold supersaturation will occur where Au metal NPs initially form from a sulfide-free hydrothermal fluid due to cooling and acidification of the H_2_O-CO_2_ hydrothermal fluid followed closely by rapid decompression. This is considered to be an early Au NP pre-concentration mechanism and we show that metal NPs may remain in solution in association within colloidal silica. At the site of mineralisation, the subsequent rapid decompression will result in H_2_S loss from the H_2_O-bearing liquid and its escape along with the CO_2_-rich vapour^48^. The precipitation of much of the remaining gold, along with silica and carbon, result from a rapid change in ƒCO_2_. We propose that these rapid changes in fluid chemistry are due to changes in the behaviour of the H_2_CO_3_ pH buffer and subsequent phase separation combined with physical changes in the hydrothermal fluid that resulted in focused deposition of Au. This efficient deposition is the result of both physical deposition of the colloidal silica/Au-NP suspension alongside the precipitation of nearly all gold remaining in solution.

This series of processes is unique to CO_2_-rich fluids, as CO_2_-poor fluids are much more susceptible to rock buffered pH control. In CO_2_-poor fluids a lack of acid-buffering by H_2_CO_3_ is antithetical to Au deposition, furthermore, raising pH due to fluid-rock interactions increases Au solubility while temperature decrease reduces Au solubility. The overall result in the latter case would be a much less focused Au deposition and lower Au ore grades. Thus CO_2_-poor fluids are more prone to gradual changes in Au solubility due to fluid-rock interactions and early formation of pyrite, yielding less focused deposition along the path towards flash vaporization.

The series of rapid changes predicted for the CO_2_-rich systems is supported by our observations of other disequilibrium features in the deposits (hydrous silica gel, amorphous carbon and metal NPs). The reactive nature of this proposed deposition mechanism highlights how feedbacks between phase separation and chemical stability of Au complexes enrich the hydrothermal fluid in Au prior to veining and Au deposition. It remains unclear whether this enrichment may be taking place at or close to (<100 m) the site of Au deposition or more distally (>100 m). Further examination of Au complex stability and transport of colloidal silica and Au NP suspension during this transition could help improve our understanding of what is required for this process to lead to focused, high-grade Au deposition.

## Method

### Sample selection

Five hand-samples of gold-rich quartz veins were obtained from five gold deposits which are characterised by locally high-grade (with abundant visible gold^49^) deposits that formed at crustal depths ranging from >5 km to 1.5 km deep (Fig. 1). The deposits formed in different host rocks and ages ranging from the Archean to the Cretaceous (Fig. 1). Details on the age of formation, pressure and temperature conditions of mineralisation emplacement, petrology and host-rocks of the deposits are summarized in SI.

### Sample preparation

For each sample, a small piece of approximately 2 cm × 2 cm containing coarse gold was cut and embedded in a one-inch epoxy mount. The sample preparation was designed to avoid surface contamination by Si and C as much as possible. The mounts were first mechanically ground (1200 grit) using AlO abrasive and then polished in three successive steps (9, 3, and 1 µm) with diamond polishing compounds. It is worth noting that carbon with diamond crystalline structure as used for fine polishing cannot be mistaken with amorphous carbon phases with the analytical techniques used in this study. The mounts were subsequently coated with platinum for SEM investigations.

### Petrographic characterisation

All the analytical work was completed at the Centre for microscopy, characterisation and analysis (CMCA) at the University of Western Australia.

Petrographic imaging and elemental analysis of the mounts were carried out using a Verios XHR SEM equipped with an Oxford Instrument 80 mm^2^ X-max SDD EDS detector. This step was followed by the extraction of ultrathin TEM samples (foils) of approximately 10 × 10 × 0.1 µm, designed to investigate the microscopic inclusions within the gold grains using a dual-beam FIB-SEM system (FEI Helios NanoLab G3 CX). Electron beam imaging within the dual-beam FIB was used to identify previously mapped areas of interest in the mounts allowing site-specific TEM samples to be prepared. The TEM sections were prepared through a series of steps involving different ion beam energies (2–30 kV) and currents (40 pA–21 nA). After initial thinning to ~1 μm the foils were extracted using an in-situ Tungsten micromanipulator and welded onto PELCO FIB-lift-out Cu TEM grids. Final thinning to ~100 nm was then achieved on the grid using lower beam currents.

The TEM sections were then analysed using a FEI Titan G2 80-200 TEM/STEM with ChemiSTEM Technology operating at 200 kV. High Resolution imaging, High Angle Annular Dark Field Scanning Transmission Electron Microscopy (HAADF-STEM) imaging and Energy-dispersive X-ray spectroscopy elemental mapping were carried out to obtain structural and compositional information from the samples. The element maps were obtained by energy-dispersive X-ray spectroscopy (EDS) using the Super-X detector on the Titan in STEM mode with a probe size ~1 nm and a probe current of ~0.9 nA. Element concentrations were calculated from EDS spectra using optimised procedure for thin films by Cliff–Lorimer quantification method built in the Bruker Esprit software. Details on the areas selected for each measurement are provided in SI.

## Supplementary information

Supplementary information

## Data Availability

The data generated in this study are provided in the Supplementary Information and the raw data are available under accession code https://research-repository.uwa.edu.au/en/datasets/nanoparticle-suspensions-from-carbon-rich-fluid-make-high-grade-g.
