# Supplementary material for: Nanoparticle suspensions from carbon-rich fluid make high-grade gold deposits
Source: Nat Commun. 2022 Jul 1;13:3795. doi: 10.1038/s41467-022-31447-5 (PMC9249814; doi:10.1038/s41467-022-31447-5)
Supplement: Supplementary file 1 — Supplementary information [file 41467_2022_31447_MOESM1_ESM.pdf]

Nanoparticle suspensions from carbon-rich fluid make high-grade gold deposits

Laura Petrella[1*], Nicolas Thébaud[1], Denis Fougerouse[2], Brian Tattitch[1], Laure Martin[3], Stephen Turner[4], Alexandra Suvorova[3], Sarah Gain[5]

[1]Centre for Exploration Targeting, University of Western Australia, Crawley 6009, Australia

[2]School of Earth and Planetary Sciences, Curtin University, Bentley 6102, Australia

[3]Centre for Microscopy Characterisation and Analysis, The University of Western Australia, Perth, Western Australia, Australia

[4] Newmont Corporation, Welshpool, WA 6106, Australia

[5] Geological Survey of Western Australia, Perth, Western Australia, Australia

[*]*Corresponding author:* laura.petrella@uwa.edu.au

Supplementary information

Table of Contents

1 Descriptions of Deposits and Samples 3
1.1 Red Lake sample (Balmer, Ontario, Canada) 3
1.1.1 Deposit details 3
1.1.2 Sample description 3
1.2 Beta Hunt sample (Kambalda, Western Australia, Australia) 4
1.2.1 Deposit details 4
1.2.2 Sample description 4
1.3 Discovery sample (Yellowknife, Northwest Territories, Canada) 5
1.3.1 Deposit details 5
1.3.2 Sample description 6
1.4 Callie sample (Granites-Tanami Gold Province, Northern Territory, Australia) 6
1.4.1 Deposit details 6
1.4.2 Sample description 7
1.5 Sixteen to One sample (Alleghany District, Sierra County, California) 7
1.5.1 Deposit details 7
1.5.2 Sample description 8
2 Investigation by Transmitted Electron Microscopy (TEM) 9
2.1 Red lake sample 9
2.2 Beta Hunt sample 9
2.3 Discovery sample 9
2.4 Callie sample 9
2.5 Sixteen to One sample 10
3 Quantitative STEM-EDS measurements 17
3.1 Beta Hunt sample 18
3.1.1 Foil 3 18
3.1.2 Foil 2 18
3.2 Discovery sample 18
3.2.1 Foil 1 18
3.2.2 Foil 2, area 1 19
3.2.3 Foil 2, area 2 19
3.3 Red Lake sample 20
3.4 Callie sample 21
3.5 Sixteen to One sample 21
3.6 Amorphous carbon analysis 22
References 23

1 Descriptions of Deposits and Samples

A summary of the characteristics of each deposit part of this study is presented in Table 1 and further details on each deposit is presented hereafter.

Table 1 – List of the deposits included in this study including the mineralisation age, location, type of host-rock and mineral assemblages. Au: gold, qz: quartz, apy: arsenopyrite, py; pyrite, po: pyrrhotite, bio : biotite, cb : carbonate, mg: magnetite, cpy: chalcopyrite, sp: sphalerite, ch: chlorite, sr: sericite, gr: graphite, mar: mariposite ($K(Al,Cr)_2(Al,Si)_4O_{10}(OH)_2$), tet: tetrahedrite ($Cu_6(Cu_4C_2)Sb_4S_{12}S$).

Deposit Name	Location	Age	T(°C) of ore formation	P(°MPa) of ore formation	Host-rock	Ore petrology	References
Red Lake	Superior Province, ON, Canada	between c. 2723 and 2712 Ma	250 to 390	> 120	metatholeitic and meta-komatiitic basaltic rocks	Au, qz, apy, py, po, bio, cb ±mg trace of cpy, sp	Dubé et al., 2004; Chi et al., 2003
Beta Hunt	Kambalda-St Ives region, WA, Australia	between ~2675 Ma and ~2640Ma *	285 to 350	80 to 140	Metasedimentary and metabasaltic rocks	Au, py, qz, cb, po, ch, mg	Veilreicher et al., 2015, Thebaud et al., 2018
Discovery	Slave Province, NWT, Canada	c. 2591 Ma *	180 to 360*	100 to 200*	Metavolcanic and metasedimentary rocks	Au, qz, apy, po, cpy, gn, sp, sr, cb	Hansen, 2013; Ootes et al., 2011
Callie	Granites-Tanami Gold Province, NT, Australia	c. 1805 Ma	209 to 404	70 to 130	Metasedimentary rocks (decarbonized siltstones)	Au, qz, bio, ch, py, cb, po	Petrella et al., 2020b; Menhard et al., 2007
Sixteen to One	Sierra Foothills Gold Province, CA, USA	c. 115 Ma	200 to 300*	37 to 350*	Metasedimentary and ultra-mafic rocks	Au, qz, po, py, apy, sp, cpy, gn, cb, ch, sr, gr, mar, tet	Marsh et al., 2008; Bierlein et al., 2008; Coveney, 1981

* data obtained from contemporaneous deposit(s) from the same greenstone belt

1.1 Red Lake sample (Balmer, Ontario, Canada)

1.1.1 Deposit details

Red Lake is a producing gold mine that is part of the Mesoarchean Red Lake greenstone belt located in the Superior Province, Western Ontario, Canada. Campbell-Red Lake is a world-class deposit with a total production of approximately 840 tons of gold and characterized by exceptionally high-grade mineralisation with previous average gold grades up to 88 g/t Au [1,2]. The high-grade mineralisation is mainly hosted in tholeiitic basalt locally intercalated with komatiitic basalt [3] which have been metamorphosed to middle greenschist and amphibolite facies [4]. The mineralisation occurs in iron-carbonate - quartz veins and breccias that host native gold, fine arsenopyrite, disseminated pyrite, pyrrhotite, magnetite and traces of fine-grained chalcopyrite and sphalerite [2,3]. Gold mineralisation occurs within quartz veins estimated to have been emplaced at temperatures that range from 350° to 550°C and pressures up to 257 Mpa based on fluid inclusion studies on quartz related to mineralisation [1,5], corresponding to a depth close to 9 km deep. An accurate estimate of the mineralisation emplacement depth is precluded by uncertainty on fluid temperature but [5] study suggests a depth of mineralisation emplacement > 5km deep. The high-grade mineralisation is interpreted to have formed between ca. 2723 and 2712 Ma based on U-Pb geochronologic data combined with detailed mapping and crosscutting relationships [2].

1.1.2 Sample description

The sample selected for this study is a gold-rich quartz vein from the Red Lake mine (Figure 1A). The three main components of the sample are, in order of abundance: quartz, visible gold and arsenopyrite. The quartz is crystalline and fine-grained, and the gold is coarse-grained and associated with fine-grained arsenopyrite.

Electron microscopy backscatter images show that gold is either associated with euhedral arsenopyrite (between 5 µm to 0.5 mm) or, to a lesser extent, pyrite and pyrrhotite (Figure 1B, C). Gold occurs as free coarse grains in the quartz veins but also forms rims around arsenopyrite and is found in micro-fractures in arsenopyrite. Berthierite ($FeSb_2S_4$) is associated with gold and arsenopyrite. Inclusions, present in the gold grains, contain silica and/or carbon. These inclusions appear sub-rounded at the surface of the sample, represent < 1 vol. % of the quartz grains and are under 3 µm in diameter (Figure 1D).

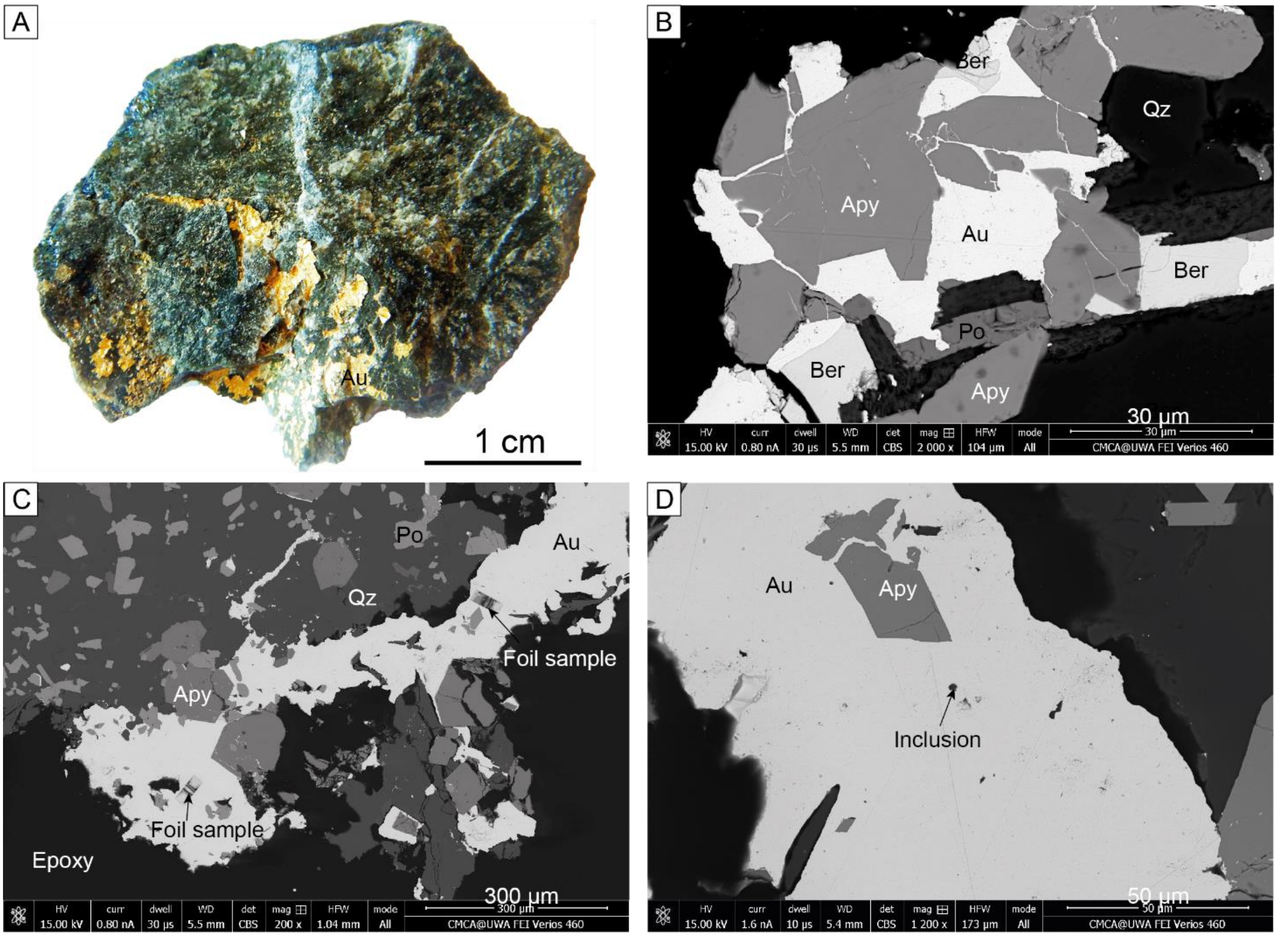

Figure 1: **A.** Photograph of the gold-rich quartz vein sample studied from the Red Lake mine; **B.** Backscatter electron image showing the textural relationship between quartz (Qz), arsenopyrite (Apy), berthierite (Ber), pyrrhotite (Po) and gold (Au); **C.** Backscatter electron image showing the interstitial texture of gold in relationship to pyrrhotite and arsenopyrite and showing the location of the two FIB samples collected for TEM analysis; **D.** Backscatter electron image showing a coarse gold grain and a micro-inclusions in gold.

1.2 Beta Hunt sample (Kambalda, Western Australia, Australia)

1.2.1 Deposit details

Beta Hunt is a producing nickel and gold mine that is located in the Kambalda-St Ives region in Western Australia within the central portion of the Norseman-Wiluna greenstone belt [6]. The Beta Hunt mine is atypical for hosting two genetically-unrelated types of mineralisation in different parts of the mine: komatiite-related massive sulfides and orogenic-style gold locally preserving exceptional high-grade intercepts containing up to 20% Au [7]. At Beta Hunt, gold mineralisation is found in quartz veins intersecting meta-basalt intercalated with graphitic metasedimentary rocks metamorphose at greenschist facies [8,9]. The gold mineralisation is believed to have been emplaced at mid-crustal conditions; temperature and pressure were estimated from fluid inclusion studies and returned temperatures that range from 285 to 350°C and fluid pressures that range from 80 to 140 MPa, corresponding to a depth of formation greater or equal to 3 km deep [9,10]. No direct dating exists for the Beta Hunt deposit, however, age of mineralisation is estimated to have occurred between ~2675 Ma and ~2640Ma based on nearby dated gold mineralisation [11,12].

1.2.2 Sample description

The sample studied (SLM191122-001) is a gold-rich quartz vein from the Beta Hunt mine (Figure 2A). At a macroscopic scale, two main components can be identified which are, in order of abundance: quartz and coarse gold. The quartz is crystalline and coarse-grained varying between 2 mm to 1 cm in diameter. The gold and carbonaceous material are both interstitial to the quartz crystals. Consequently, the shape of the gold grains follows the shape of the outer boundary of the quartz crystals. Minor dark telluride-minerals are also locally associated to gold in the sample. Gold is also found in secondary fractures within the quartz.

At a microscopic scale, the interstitial texture of the gold can be easily identified on the backscatter electron images of the sample (Figure 2B). The backscatter images reveal the presence of inclusions within the gold grains, which vary from less than 1 µm to 3 µm in diameter. Only limited number of the inclusions appear to contain silica phase. Three inclusions were targeted in a gold grain due to their larger size and because their EDS spectra indicated the presence of silica (Figure 2C).

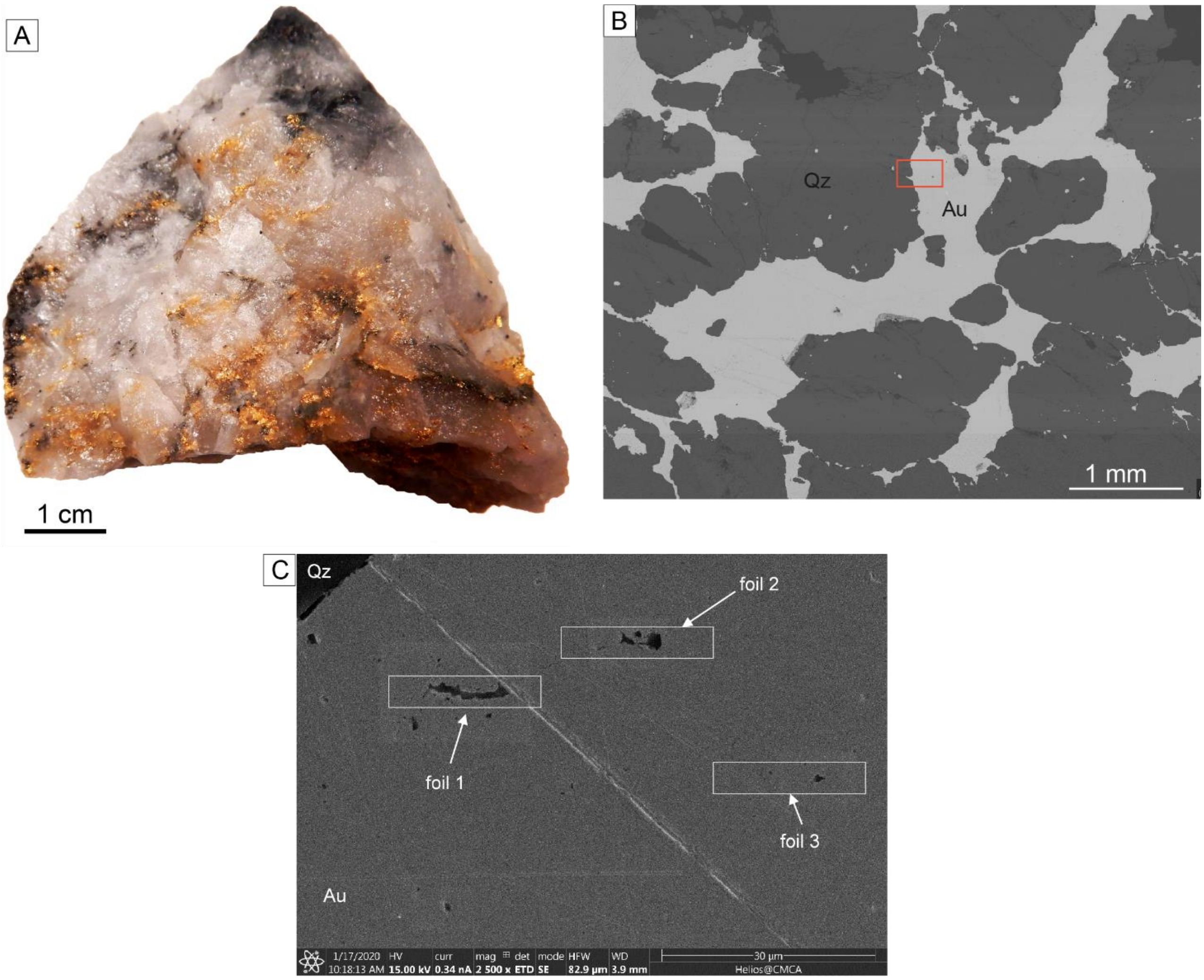

Figure 2: **A.** Photograph of the sample studied: quartz vein rich in visible gold containing lesser amounts of carbonaceous material. **B.** Backscatter electron image (BSE) showing the textural relationship between gold and quartz. Micrometric inclusions are present in gold grains (dark spots in gold crystals). The red rectangle shows the area in which the foils were extracted. **C.** Backscatter electron image of area in red rectangle in B, showing the inclusions in gold targeted in this study and the location of the three foils extracted from the sample. Au: gold, Qz: quartz.

1.3 Discovery sample (Yellowknife, Northwest Territories, Canada)

1.3.1 Deposit details

Discovery is a past producing mine located in the Slave Province in the Northwest Territories of Canada in the northern part of the Yellowknife Greenstone Belt. The Discovery mine has produced ~1 million ounces [13] of mostly high-grade visible gold in quartz veins, hosted in highly silicified and sulfidized metasedimentary rocks which were metamorphosed to the upper greenschist to amphibolite facies [14]. The quartz veins generally trend NW and dip SW [14]. The mineral paragenesis includes gold, arsenopyrite, ilmenite, biotite, pyrrhotite, chalcopyrite, galena, sphalerite and pyrite [14]. A fluid inclusion study conducted on quartz-carbonate veins related to gold at the Giant deposit, which contains similar mineralisation and is hosted within the same Yellowknife greenstone belt, estimated that mineralisation was emplaced at temperatures near 350°C and pressures that ranged from 100 to 200MPa which corresponds to depth of formation greater than 4 km deep [2,15]. The age of mineralisation is estimated at 2591 ± 37 Ma obtained from the similar, nearby Con deposit in the Yellowknife Greenstone Belt using Re-Os geochronology on pyrite associated with mineralisation [16].

1.3.2 Sample description

The sample is a gold-rich quartz vein (Figure 3A) with coarse-grained translucent, grey quartz, coarse gold grains interstitial to the quartz grains, biotite, pyrrhotite, galena and carbonaceous material, which are also interstitial to the quartz grains.

At a microscopic scale, the gold is interstitial to the quartz grains and is also found within micro-fractures in quartz. Pyrrhotite, galena and minor chalcopyrite are intergrown with gold and interstitial to the quartz grains (Figure 3B). Gold contains numerous sub-rounded carbon-rich inclusions (identified by EDX), ~ 1 vol.%, which are generally around 2 µm in diameter but can be as much as 70 µm long (Figure 3C). Different gold habits are present in this sample; gold occurs in equilibrium with quartz and also within biotite cleavages and in inclusions in pyrrhotite (Figure 3 B,C and D).

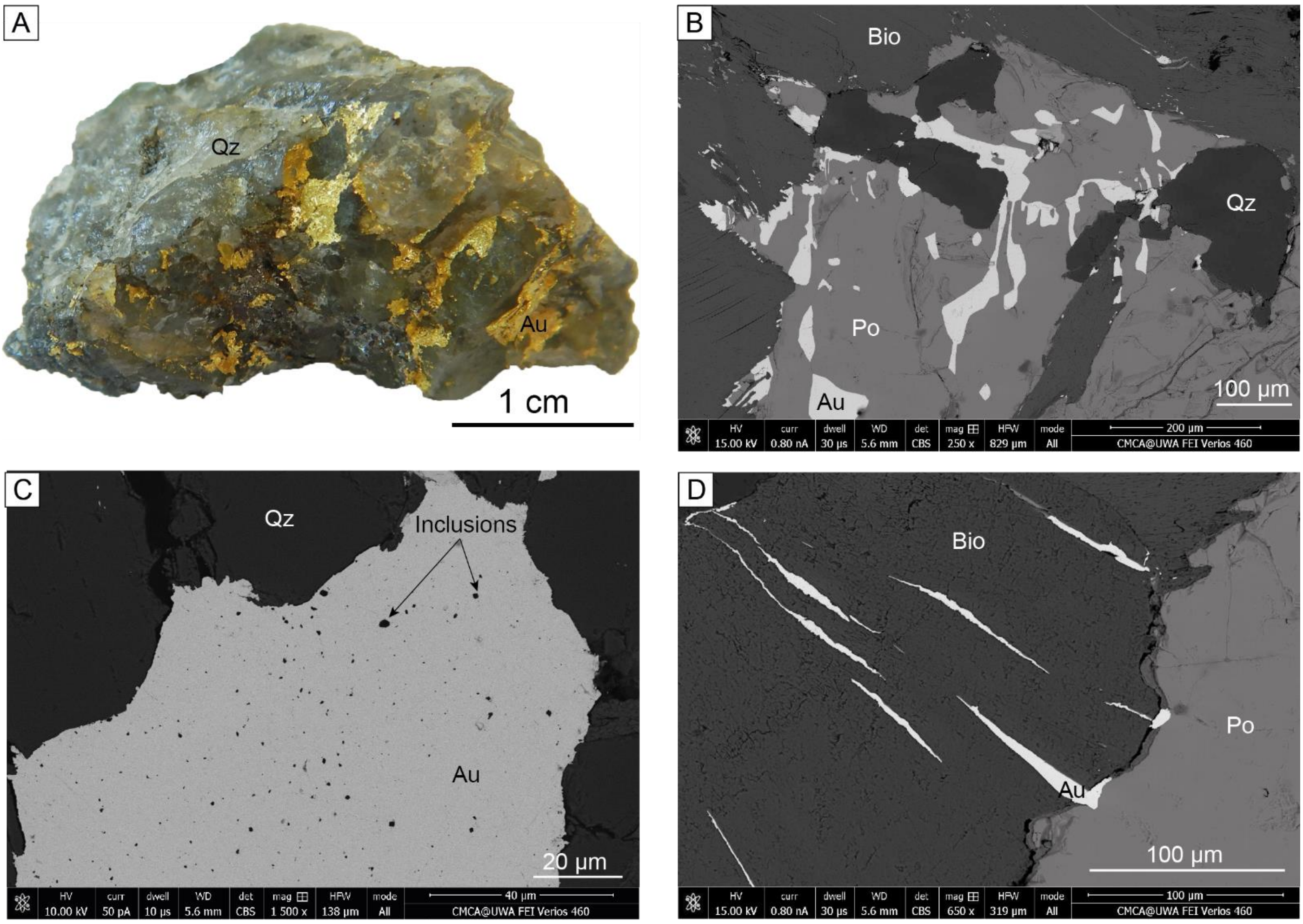

Figure 3: **A**. Photograph of the gold-rich quartz vein sample from the Discovery mine used in this study; **B.** Backscatter electron image showing the textural relationship between quartz (Qz), pyrrhotite (Po), biotite (Bio) and gold (Au); **C.** Backscatter electron image showing gold interstitial to quartz and micro-inclusions present in the gold grains; **D.** Backscatter electron image showing gold located within biotite cleavages.

1.4 Callie sample (Granites-Tanami Gold Province, Northern Territory, Australia)

1.4.1 Deposit details

Callie is a producing mine that is located in the Granites-Tanami Gold Province in the Northern Territory of Australia; it is world-class deposit with a current total endowment of c. 14.2 M oz [17]. The mineralisation is characterized by high-grade visible gold hosted in quartz veins that can contain up to 10,000 g/t of gold [17]. The high-grade mineralisation is hosted in NE-trending, SE-dipping veins interacting with finely laminated, decarbonized siltstones which were metamorphosed up to amphibolite facies. The mineral paragenesis associated with mineralisation consists of quartz, plagioclase, chlorite, biotite, calcite, traces of sulfides (pyrite, chalcopyrite, arsenopyrite and galena), gold, ilmenite, rutile, monazite, apatite and xenotime [18]. A fluid inclusion study conducted on mineralised

quartz veins at the Callie deposit estimated that the veins were emplaced at pressures that range from 70 to 130 Mpa and temperatures that range from 209 to 404°C [19], which corresponds to a depth of formation greater or equal to 3 km deep. The mineralisation was dated at c. 1805 using U–Pb geochronology on hydrothermal xenotime associated with gold [18].

1.4.2 Sample description

This sample was previously studied and results are published in Petrella, et al. [20]. This study consists in a re-analyses of the same foil used in Petrella, et al. [20]. The sample is a high-grade (~1,000 ppm Au) gold vein hosted in a decarbonized metasedimentary rock (Figure 4A). The coarse gold grains are interstitial to quartz and contain micro-inclusions less than 3 µm, Au NP were identified in one of the inclusion extracted from the Callie gold (Figure 4B,C).

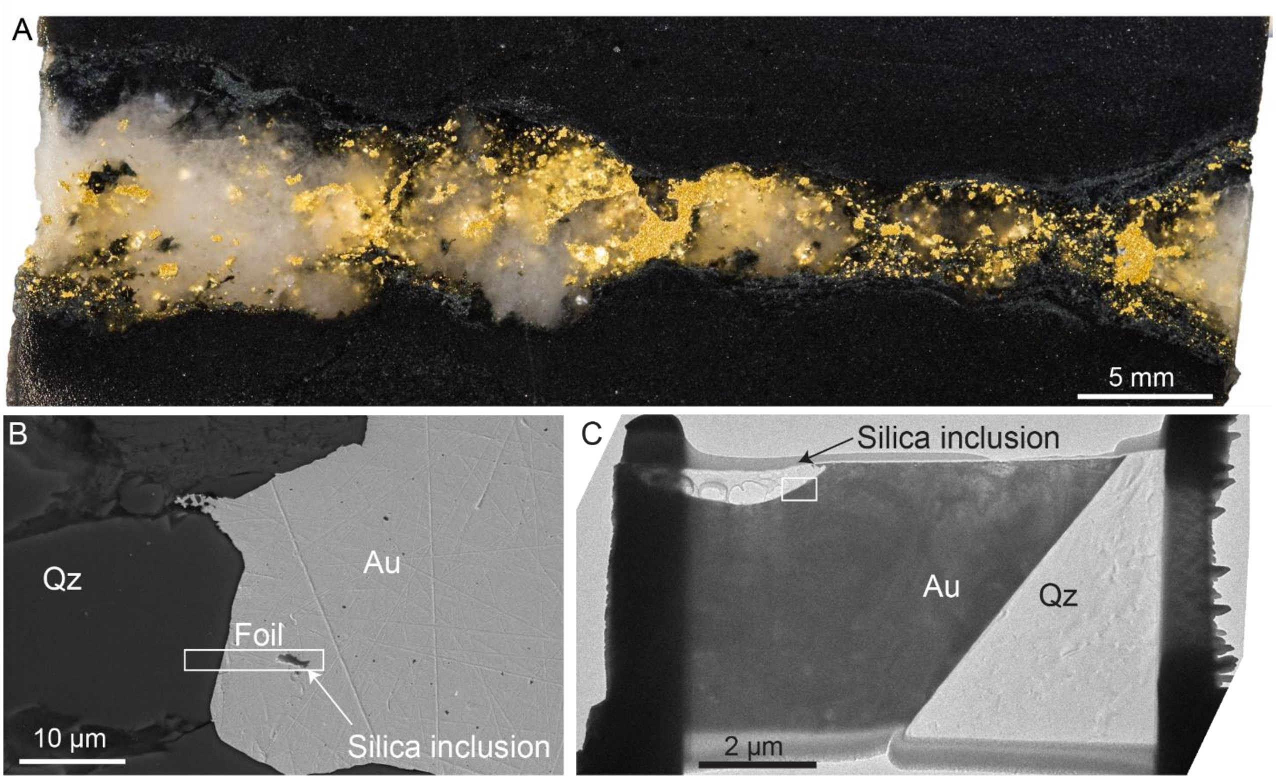

Figure 4: Modified after Petrella, et al. [20]. **A.** Photograph of the very high-grade quartz vein sample from the Callie mine used in this study; **B.** Backscatter image of the area selected showing a gold grain interstitial to quartz. The white rectangle shows the section collected for TEM analyses (foil) including gold, quartz and a silica inclusion. **C.** Scanning transmission electron microscopy (STEM) image of the foil section; the rectangle corresponds to the area where the Au nanoparticles are located.

1.5 Sixteen to One sample (Alleghany District, Sierra County, California)

1.5.1 Deposit details

The Sixteen to One deposit is a past producing mine situated in the Alleghany district within the Sierra Foothills gold province in the central Sierra Nevada Foothills Metamorphic Belt in California, USA. The gold deposits in the Sierra Foothills are structurally controlled and are described as orogenic-type mineralisation [21]. The Alleghany district produced approximately 34.3 tons of very high-grade visible gold [22] hosted in quartz veins [23]. The mineral assemblage at the Sixteen to One deposit consists of free gold in quartz veins associated with pyrrhotite, pyrite, arsenopyrite, sphalerite, chalcopyrite, tetrahedrite, and galena, as well as ankerite, mariposite, and graphite [24,25]. A fluid inclusion study on similar gold deposits in the Allegany district estimated that mineralisation was emplaced at temperatures ranging from ~200 to 300°C and pressure that ranges from ~37 to 250 MPa [26] which corresponds to a depth of formation greater or equal to 1.5 km deep. The mineralisation is mainly hosted in E-dipping veins associated with N-S trending first-order structures [23] which intersect Carboniferous metasedimentary rocks and serpentinized ultramafic dikes [24]. Mineralisation was dated at 114.7 ± 1.4 Ma using $^{40}Ar/^{39}Ar$ geochronology on mariposite (Cr-rich muscovite) [25].

1.5.2 Sample description

The sample is from a gold-rich quartz vein (Figure 5A) and consists of white, coarse-grained quartz, with coarse gold grains, carbonaceous material, arsenopyrite, galena and feldspars.

Electron microscope investigation shows that gold occurs as free grains interstitial to quartz or associated with arsenopyrite as rims or within micro-fractures (Figure 5B). Numerous (up to 1 vol.% of the gold) sub-rounded carbon or silica inclusions are present in the gold grains, generally ~ 2µm in size (Figure 5B).

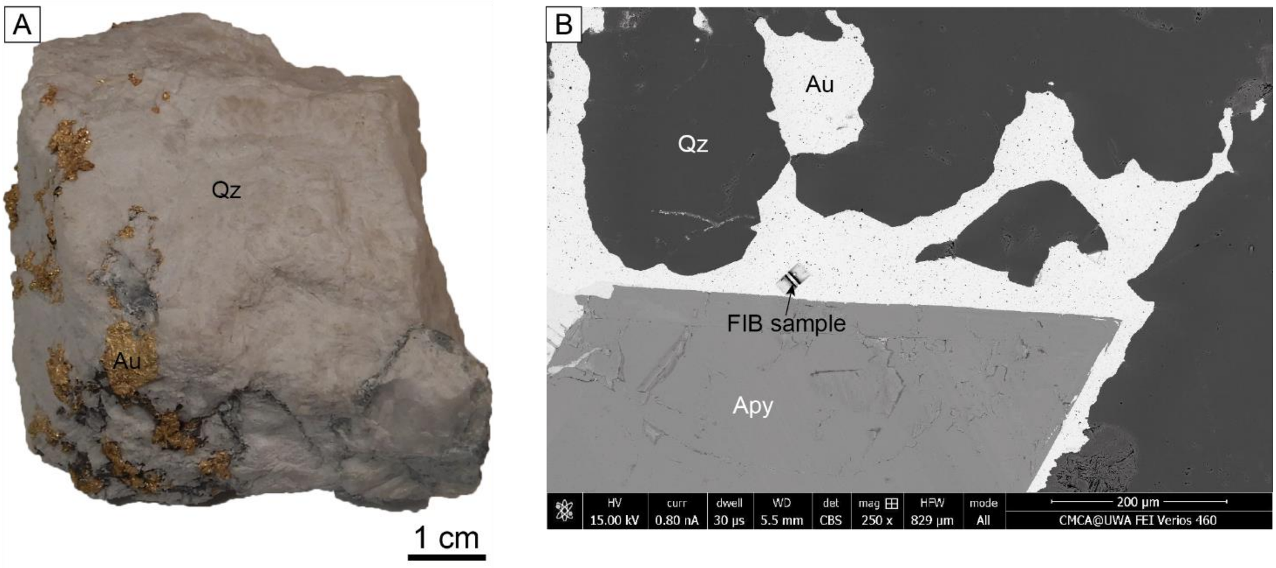

Figure 5: **A.** Photograph of the gold-rich quartz vein sample studied from the 16 to 1 mine; **B.** Backscatter electron image showing the textural relationship between quartz (Qz), arsenopyrite (Apy) and gold (Au). The black dots in the gold are micro-inclusions of carbon and/or silica. The image also shows the location the foil collected for TEM analysis.

2 Investigation by Transmitted Electron Microscopy (TEM)

For TEM investigation, thin samples (foils) were carefully selected and extracted from selected gold grains in each sample. This study is focused solely on coarse gold grains and gold that appears to be late or remobilised was not included. The foil location was determined by the presence of inclusion in gold at the surface of the sample because in our previous study NPs were found preserved in inclusions in gold grains.

The summary of the inclusions and NPs identified by this study is provided in the Table 2 below.

Table 2: Summary of the composition of the micro-inclusions and the NPs identified in this study for each deposit.

Deposit	Inclusion No	Inclusion composition	NP composition	No of NP	Size of NP (nm)
**Red Lake**	Inclusion 1	amorphous carbon	Electrum	>50	2 to 20
**Beta Hunt**	Inclusion 3	amorphous carbon and silica	Gold	>10	3 to 5
**Discovery**	Inclusion 1	amorphous silica	Gold	4	5 to 8
	Inclusion 2	amorphous carbon	Silver oxide	~20	8 to 15
**Callie**	Inclusion 1	amorphous carbon and silica	Gold	>20	1 to 11
**Sixteen to One**	Inclusion 1	crystalline carbon	Copper	>20	20 to 100

2.1 Red lake sample

Free gold grain in equilibrium with quartz was selected for TEM analysis from the Red Lake sample. The gold grains which texture suggests a late deposition or local remobilisation of the gold, such as gold hosted in sulphide micro-fractures, were not integrated in the TEM study. Further details on the studied inclusion location and composition are included in Figure 6. EDS element maps show the distribution of selected elements in the samples. Please note that individual element maps might locally show artefact in gold (presence of elements within the gold that are not there). There are three problems causing the artefacts: 1) in some areas two phases of different composition overlap; 2) there is a peak overlap between Au, Ag and C in lower energy region, and 3) there is a strong scattering due to Au phase presence.

2.2 Beta Hunt sample

The gold grain investigated is interstitial to and in equilibrium with quartz, gold located in fractures cross-cutting the quartz veins were not considered for this study. Three inclusions in one gold grain were extracted for TEM analysis (Figure 2 C). Gold NP were observed in an amorphous silica phase within the upper part of one inclusion (Figure 7). The NPs are sub-rounded to elongated and the largest is ~ 20nm long, other smaller NPs size is below 5 nm (Figure 7D)

2.3 Discovery sample

Two foils were extracted from the Discovery sample in coarse gold grain in equilibrium with quartz (Figure 3 C), gold in inclusion in pyrrhotite and biotite was included in the study.

Foil 1: Further details on foil 1 location and composition is included in Figure 8. The EDS element maps of the inclusion indicates that the amorphous phase is composed of silica and the four NPs encapsulated in the amorphous silica are composed of Au (supplementary information)

Foil 2: The composition of each phase in foil 2 is confirmed by EDS element maps and the crystalline state is confirmed by HRTEM and FFT diffractograms (Figure 9).

2.4 Callie sample

This study presents a re-analyse of a foil from the Callie sample presented in our previous work [20]. Further details on the foil are included in Figure 10.

2.5 Sixteen to One sample

One foil targeting one inclusion was extracted from a coarse gold grain (Figure 5B) from the Sixteen to One sample and further details on the inclusion composition are included in Figure 11 and Figure 12.

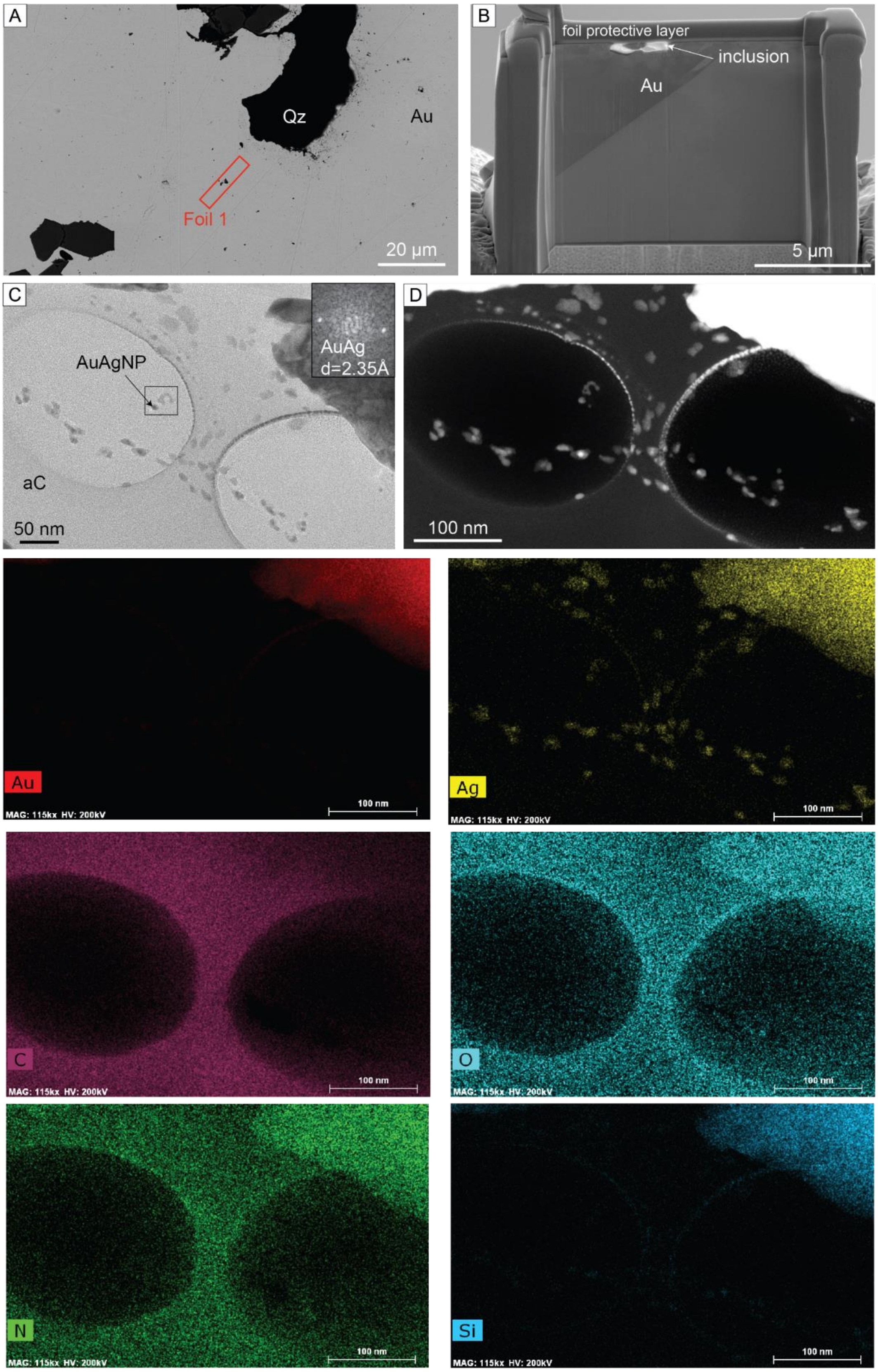

Figure 6: **Red Lake sample**. **A.** Backscatter electron image taken within a coarse gold grain of Red lake sample showing the location where the foil was extracted. **B.** TEM image of the Red Lake foil showing the platinum (Pt) protective layer at the top of the foil, an elongated inclusion at the surface of the foil. **C.** Enlarged TEM image from within the amorphous carbonic phase showing numerous subrounded AuAg NPs**.** The diffractogram in the top right corner was obtained from the NPs in the black rectangle. **D.** High-angle annular dark field (HAADF) image from the inclusions the amorphous carbonic phase showing numerous subrounded AuAg NPs in the amorphous carbonic phase. **Following EDS**

images: EDS element map of Au, Ag, C, O, N and Si overlaying High-angle annular dark field (HAADF) image and showing the presence of electrum NP in a carbonic phase. Please note that the presence of N and Si in gold is an artefact due to a phase overlap and strong scattering due the presence of Au whereas the presence of Si in gold is also an artefact due to the scattering effect of Au.

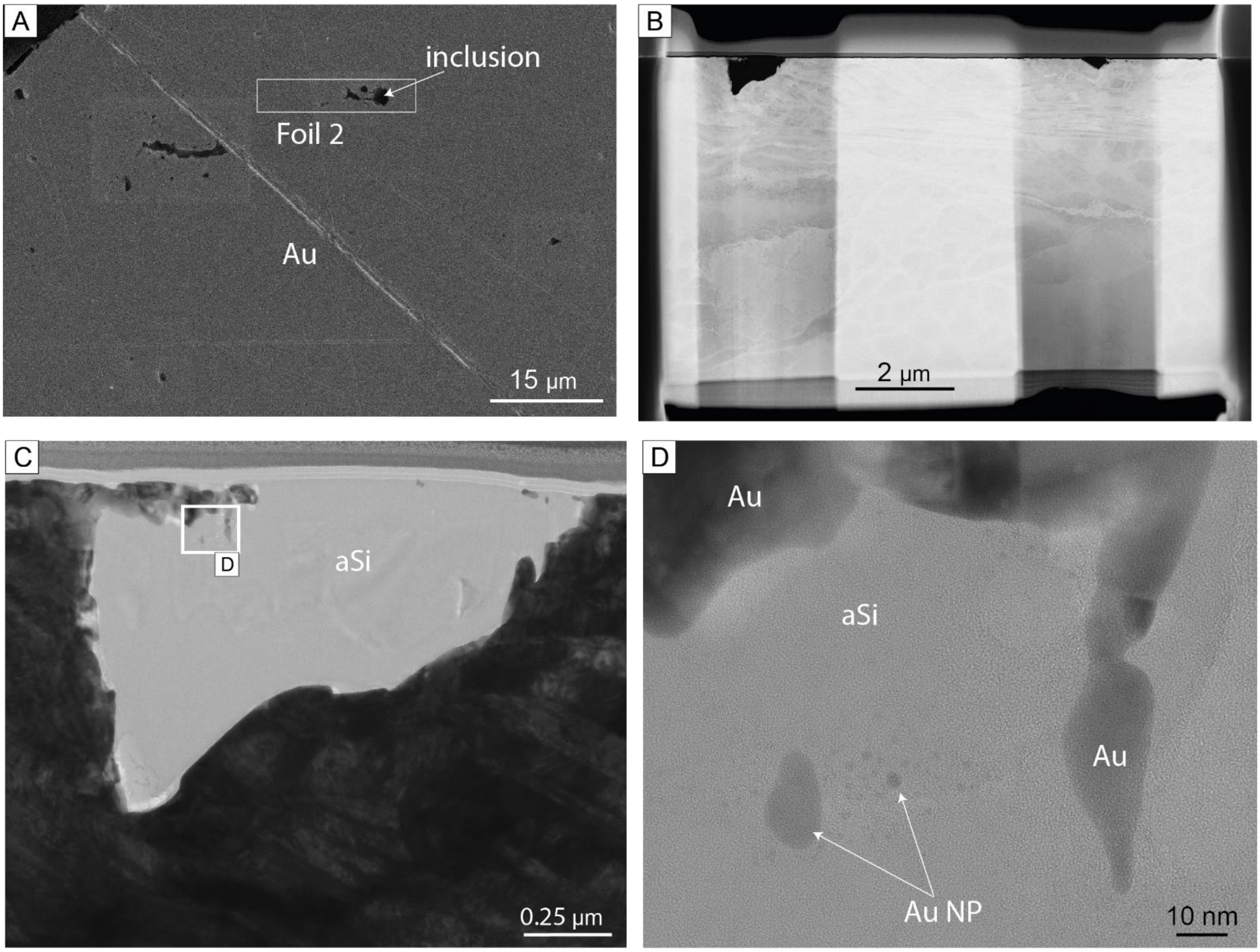

Figure 7: **Beta Hunt sample. A.** Backscatter electron image taken within a coarse gold grain and showing the location were foil 2 was extracted. **B.** STEM image of the entire Beta Hunt foil 2, showing a large inclusion on the left-hand side that was the focus of this study. **C.** TEM image of the inclusion of interest that is mainly composed of amorphous silica (aSi). **D.** TEM image showing an enlarged view of the area indicated by a white rectangle in **C.** showing a large Au NP and numerous small (<5nm) Au NPs within the amorphous silica in the upper part of the inclusion.

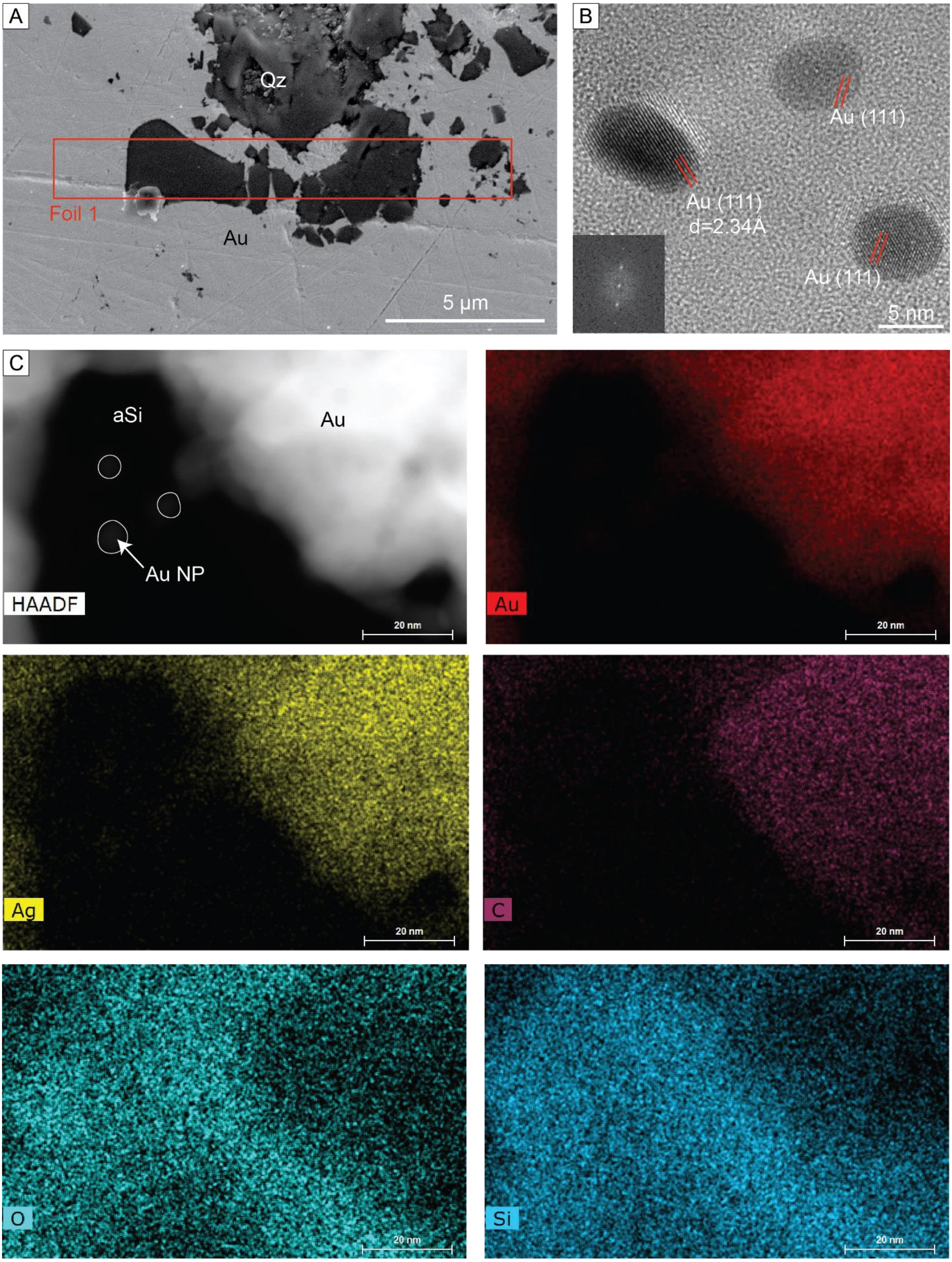

Figure 8: **Discovery sample – Foil 1. A.** Backscatter electron image showing the textural relationship between coarse gold and quartz crystals and the location were foil 1 was extracted. **B.** TEM image that shows amorphous silica phase (aSi) filling the inclusion in foil 1 and rounded Au NP floating within the amorphous phase. The diffractogram in the lower left corner was obtained from the lower left Au NP; **C.** HAADF image

of the inclusion in foil 1 showing AuNP in the middle of the image followed by EDS elemental map overlying the HAADF image showing that the inclusion is filled with silica. Please note that the presence of C in the gold is an artefact due to a peak overlap between Au and Ag N peak and C K peak.

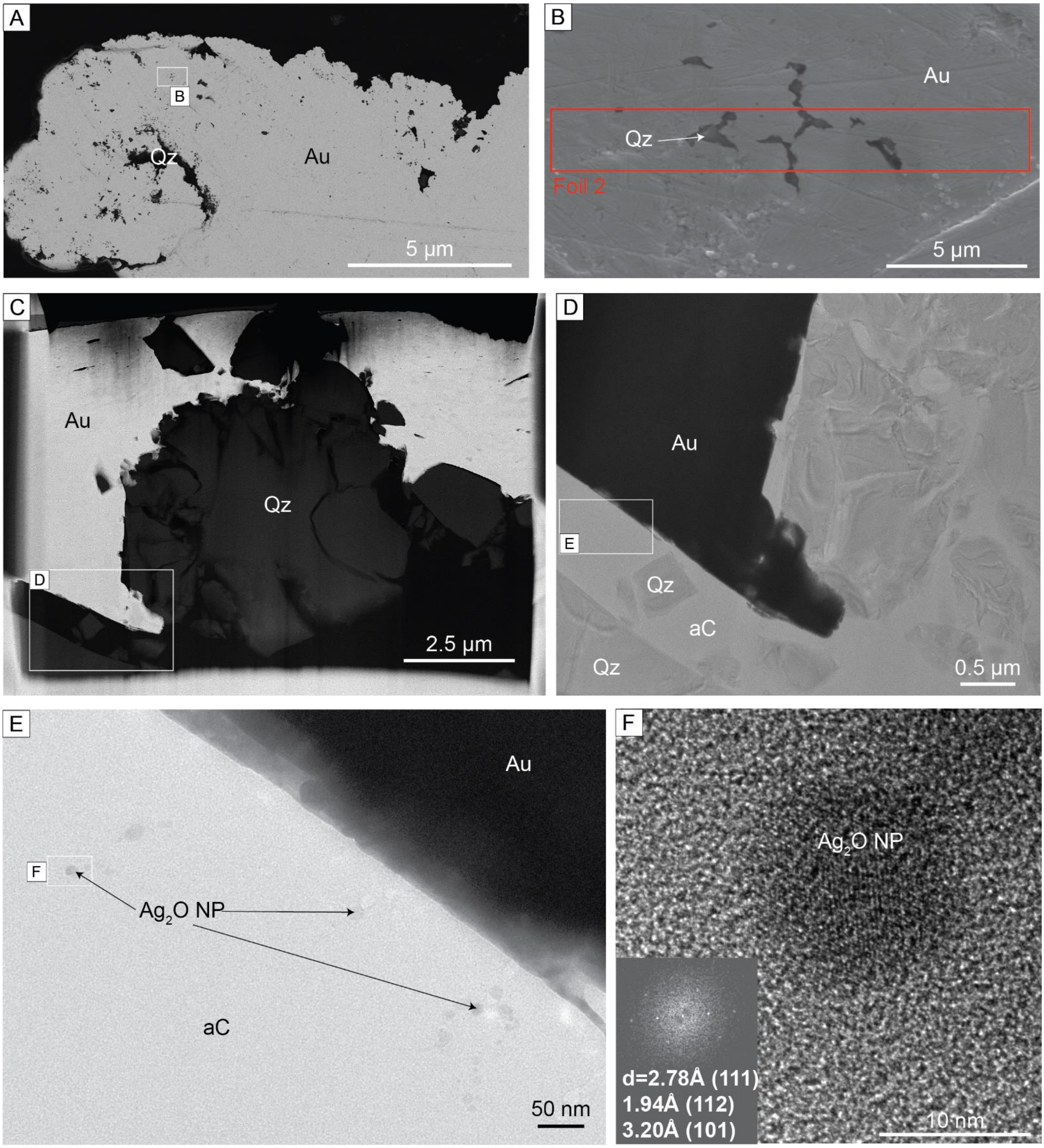

Figure 9: **Discovery sample – Foil 2. A.** Backscatter electron image from discovery sample showing the coarse gold grain from which foil 2 was extracted. **B.** Backscatter electron image taken within a coarse gold grain showing quartz inclusion and the location were foil 2 was extracted. **C.** Backscatter electron image of Discovery sample foil 2, the lighter grey crystalline phase corresponds to coarse gold (Au), the dark grey to quartz (Qz) and the black areas within the foil are composed of amorphous carbonic phase. **D.** TEM image of the area indicated by a white rectangle in C. showing the texture of fractured quartz crystals (Qz) surrounded by an amorphous carbonic phase (aC). **E.** TEM image of the area indicated by a white rectangle in D. showing rounded silver oxide nanoparticles (Ag_2O NP) within the amorphous carbon phase. The diffractogram was acquired within the carbon phase and confirms the absence of atomic arrangement. **F.** HRTEM image of the area indicated by a white rectangle in E. showing an Ag_2O NP with a measured d-spacing of 2.78Å for (111) and 1.94Å for (112) and 3.20Å for (101) that confirms its composition.

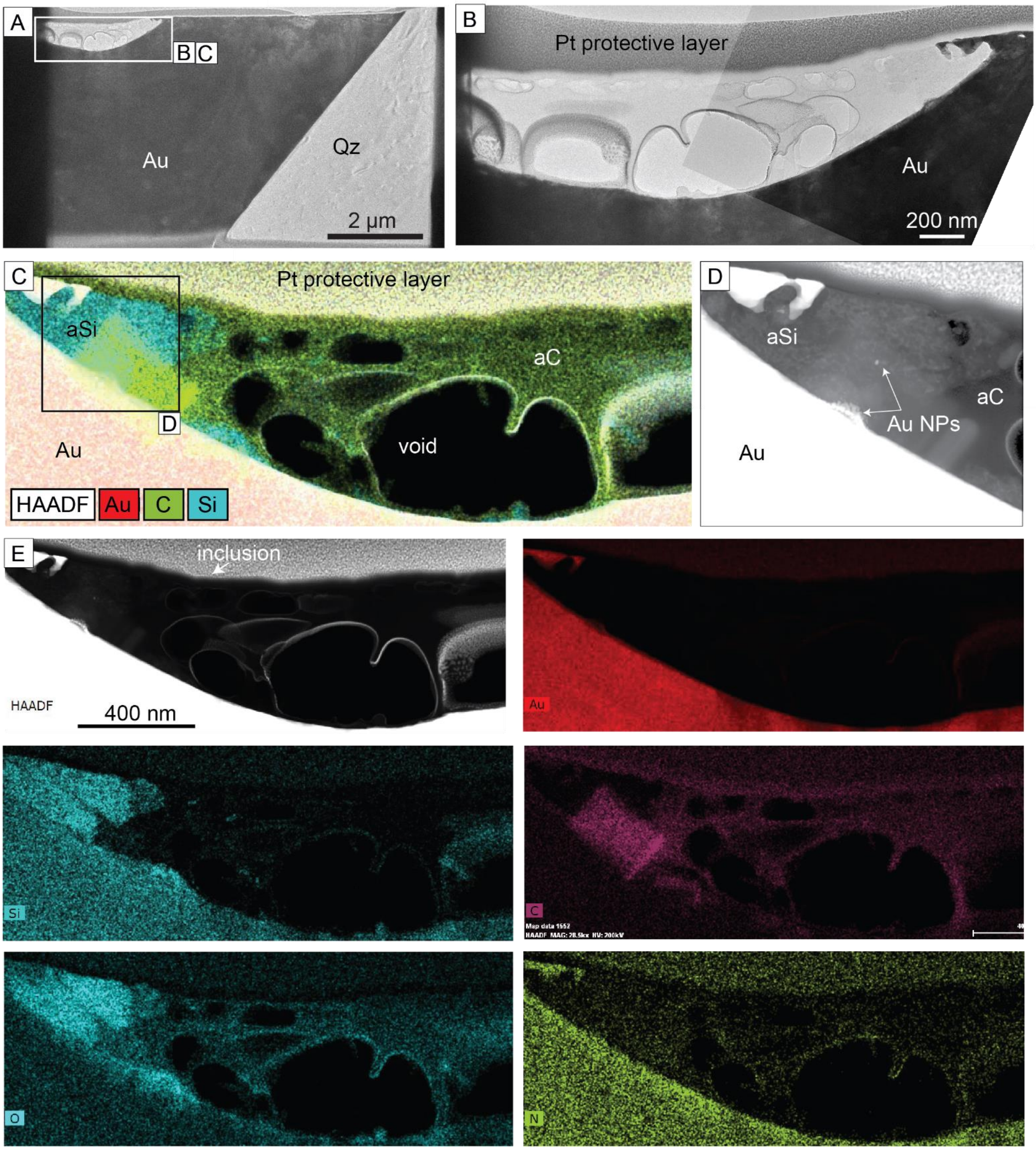

Figure 10: **Callie sample. A.** TEM image of Callie foil showing a contact between coarse gold (Au), quartz (Qz) and a micro-inclusion in gold located at the surface of the foil modified after [20]; **B.** TEM image of the area indicated by a rectangle in A, and that shows an image of the micro-inclusion composed of light grey disordered phases and voids; **C.** EDS elemental map of the Callie micro-inclusion showing that the centre and right-hand side is composed of amorphous carbon (aC) with numerous voids, and the left-hand side is composed of amorphous silica (aSi). The brighter green rectangle is an artefact caused by precedent EDS mapping over the area during previous study by Petrella, et al. [20]; **D.** HAADF image of the left-hand side of the Callie micro-inclusion showing the location of the Au NPs already reported in Petrella, et al. [20]. **E and following images.** HAADF image of the inclusion and element maps showing Au, Si, C, O and N distribution in the inclusion. Please note that the presence of N in the gold shown in the N elemental map is an artefact caused by strong scattering due the presence of the Au phase.

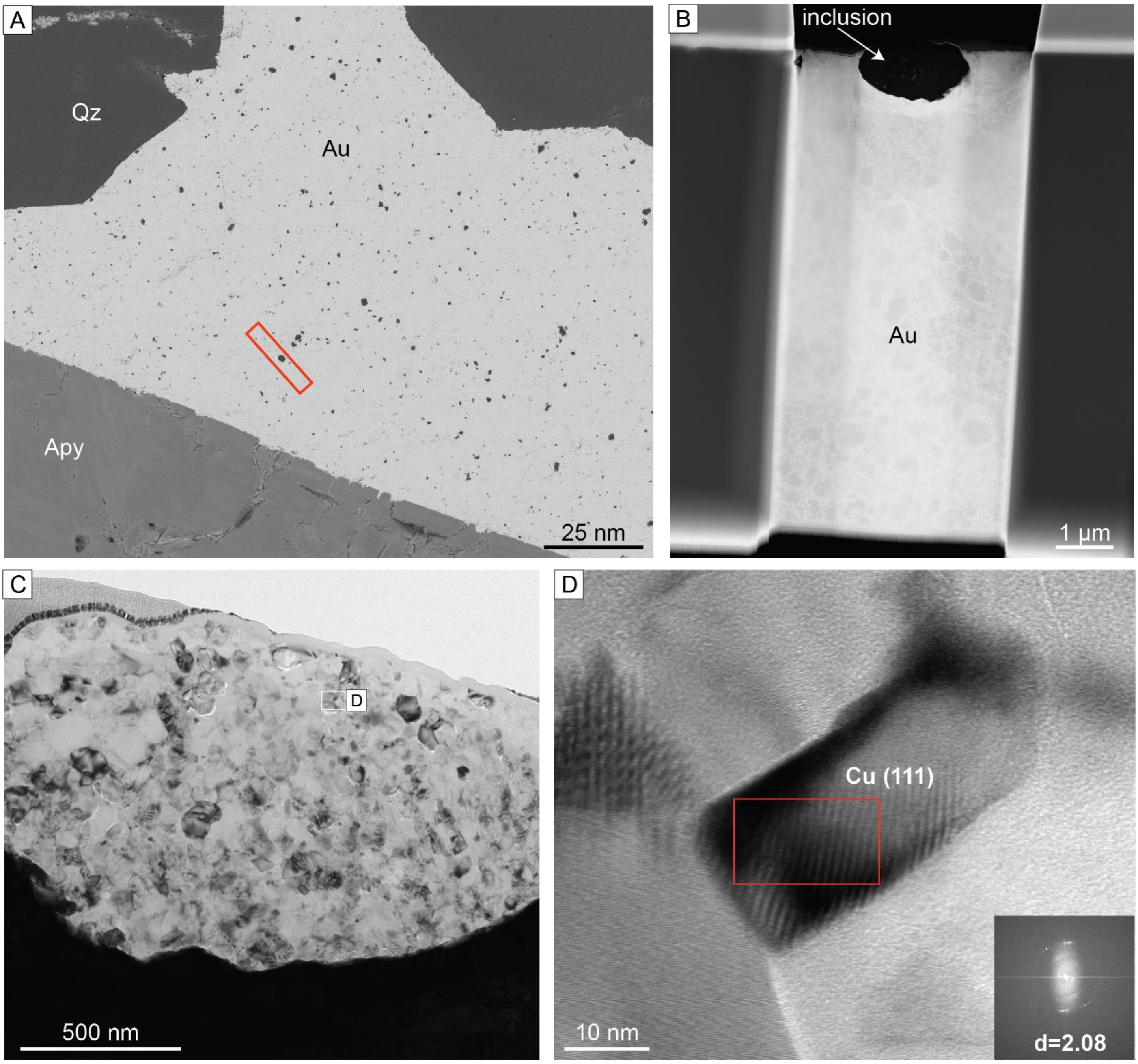

Figure 11: **Sixteen to One sample**. **A.** Backscatter electron image showing inclusions in coarse gold (Au) in equilibrium with quartz (Qz). Red rectangle shows the inclusion selected for this study; **B.** TEM image of the foil from the Sixteen to One sample showing the micro-inclusion at the surface; **C**. TEM image of the inclusion; **D.** enlarged TEM image of the area in a white rectangle in C showing a copper nanoparticles of 30 nm large and 50 nm long. and diffraction pattern indicating a d spacing of 2.08 Å that confirm the Cu nature of the NP.

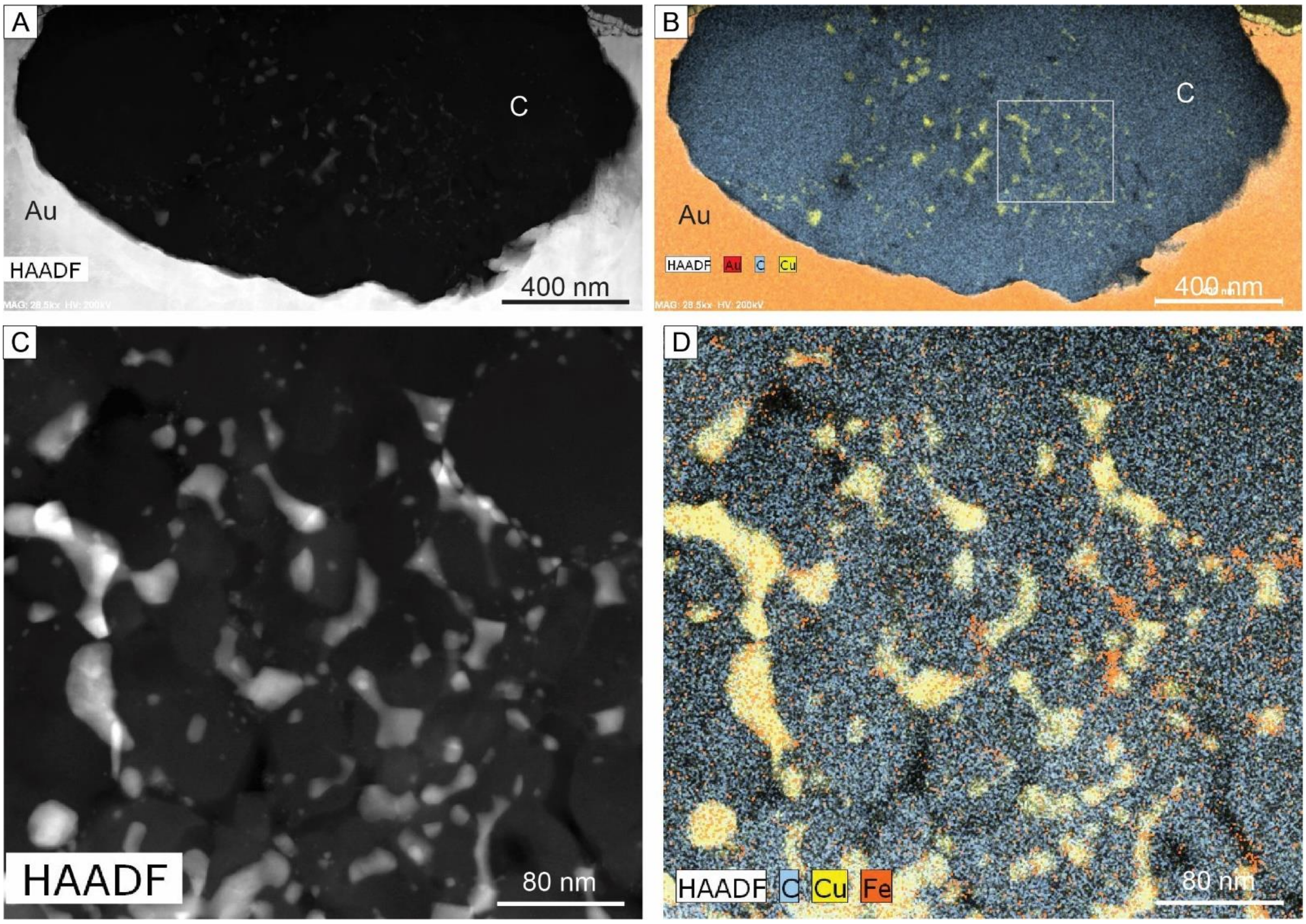

Figure 12: **Sixteen to One sample. A.** HAADF image of the inclusion; **B.** EDS elemental map of the Sixteen to One micro-inclusion showing that the inclusion is mainly composed of micro-crystalline carbon with interstitial NPs of copper; **C.** Enlarged HAADF image of the area in a white rectangle in B ; **D.** Enlarged TEM EDS elemental map of the area in a white rectangle in B showing the shape of the Cu NPs and the presence of Fe-bearing phases which have not been identified due to their small size.

3 Quantitative STEM-EDS measurements

The table below (Table 3) shows the results of compositional analysis obtained on different phases in foils from the samples studied. Cu and Ga have been excluded from the result table because the presence of both elements could be due to contamination induced by the FIB sample preparation process (note that the foil sample holder is made of Cu).

Table 3: Summary of the quantitative STEM-EDS measurement collected in this study.

Sample	Phase	O (wt.%)	O (at.%)	2 sigma	C (wt.%)	C (at.%)	2 sigma	N (wt.%)	N (at.%)	2 sigma	Si (wt.%)	Si (at.%)	2 sigma	Au (wt.%)	Au (at.%)	2 sigma	Ag (wt%)	Ag (at%)	2 sigma	Fe (wt.%)	Fe (at.%)	2 sigma	As (wt.%)	As (at.%)	2 sigma	S (wt.%)	S (at.%)	2 sigma
Beta Hunt foil 3	amorp. Silica	47.63	62.05	2.92							50.92	37.79	0.29	1.45	0.15	0.36												
	coarse gold													97.13	94.88	19.48	2.87	5.12	0.63									
	amorp. carbon and AuNP	8.17	8.34	1.26	64.95	88.30	5.74	0.71	0.83	0.41	0.72	0.42	0.37	25.45	2.11	6.70												
Beta Hunt Foil 2	amorp. silica and carbon	10.45	9.69	0.89	60.55	74.73	4.11	1.88	1.99	0.33	25.52	13.47	0.89	1.60	0.12	0.63												
	coarse gold													96.60	88.28	19.37	1.54	2.57	0.36									
	amorp. carbon	9.03	7.04	0.64	88.75	92.17	5.47	0.79	0.70	0.14				1.41	0.09	0.40	0.02	0.00	0.07									
	Fe phase	6.38	6.81	0.76	55.45	78.87	4.13	0.35	0.43	0.21				1.09	0.09	0.64				25.45	7.79	2.12				11.28	6.01	1.08
Discovery Foil 1	amorp. silica	30.23	43.66	2.03							68.28	56.17	1.19	1.49	0.18	0.54												
	coarse gold													93.68	89.03	18.79	6.32	10.97	1.32									
Discovery Foil 2_area1	amorp. silica	45.93	60.09	2.84							53.46	39.84	0.47	0.55	0.06	0.20	0.06	0.01	0.08									
	coarse gold													95.29	90.88	19.11	4.14	7.21	0.88									
	amorp. carbon and silica	18.45	20.55	1.40	32.65	48.46	2.45	3.73	4.75	0.49	40.69	25.83	1.16	4.48	0.41	1.28												
Discovery Foil 2_area2	AgO NP and amorp. carbon	7.86	7.69	1.40	67.14	87.49	6.35	1.41	1.58	0.64				2.82	0.22	1.77	20.78	3.01	5.36									
	coarse gold													95.85	92.68	19.22	4.15	7.32	0.88									
	amorp. carbon	18.30	15.61	1.22	70.63	80.24	4.42	3.24	3.16	0.32	1.02	0.49	0.14	6.47	0.45	1.45	0.15	0.02	0.12				0.19	0.03	0.12			
Red Lake	coarse gold													96.32	93.48	19.35	3.68	6.52	0.81									
	AuAg NP	0.56	5.29	0.41										70.15	53.74	16.46	29.29	40.97	7.21									
	amorp. carbon	5.64	4.42	0.41	88.75	92.65	5.41	3.03	2.71	0.25				2.49	0.16	0.57												
	amorp. carbon 1	20.72	16.55	1.54	75.80	80.63	4.94	3.06	2.79	0.52				0.39	0.03	0.24	0.03	0.00	0.09									
	amorp. carbon 2	24.05	19.34	2.54	74.22	79.52	6.14	1.21	1.11	0.68				0.53	0.03	0.41												
Sixteen to One	micro-crytalline carbon	0.16	0.12	0.08	99.61	99.86	6.13							0.18	0.01	0.13	0.05	0.01	0.08									

The quantitative measurements presented in Table 3 were extracted from specific areas in each foil. The areas selected are indicated by polygons in the EDS multi-element maps for each foil below.

3.1 Beta Hunt sample

3.1.1 Foil 3

The data extracted from Beta Hunt foil 3 includes coarse gold composition, amorphous silica (aSi) and amorphous carbonic phase (aC). The areas from where the phases composition was extracted is presented in Figure 13. Please note that the amorphous carbonic phase is thin and is overlapping with the coarse gold (Au) directly adjacent to the inclusion.

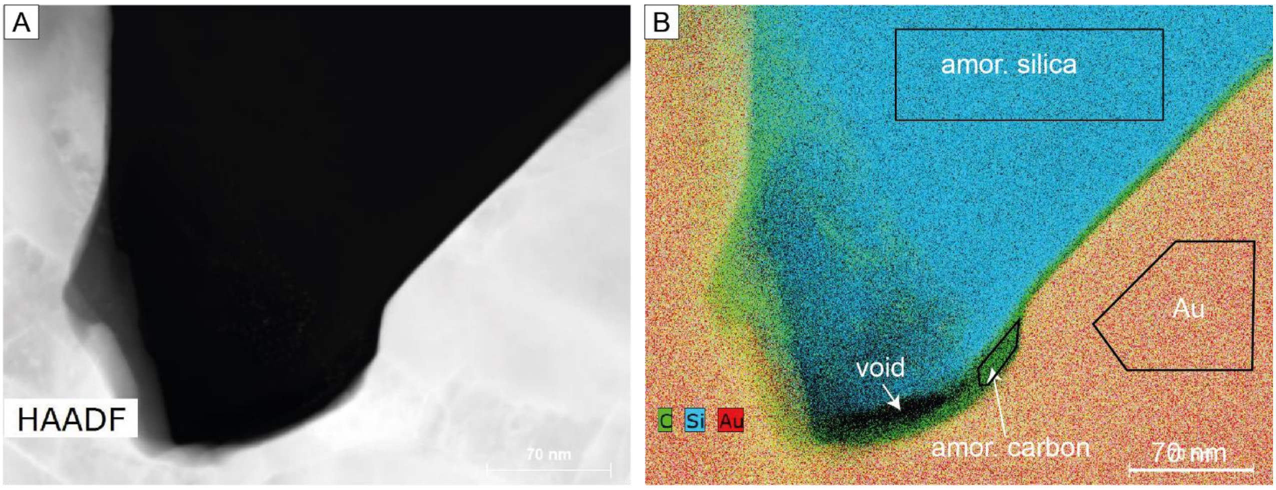

Figure 13: Beta Hunt foil 3; HAADF and multiple elements map showing the areas from which the phases composition was extracted.

3.1.2 Foil 2

The data extracted from Beta Hunt foil 2 includes coarse gold composition, amorphous silica (aSi), amorphous carbonic phase (aC) and Fe-bearing phase. The areas from where the phases composition was extracted is presented in Figure 14. Please note that here is an overlap between the amorphous carbonic phase, the amorphous silica (aSi) and the iron-bearing phase that prevents precise measurement of the composition of silica and iron phases.

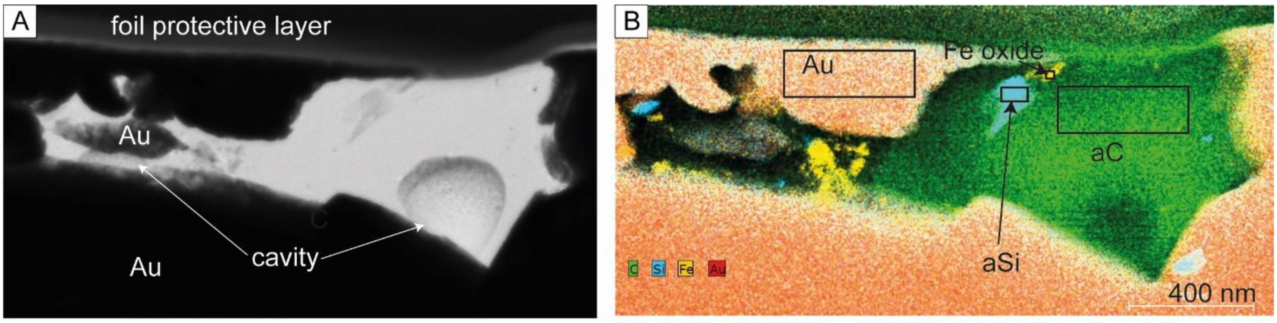

Figure 14: Beta Hunt foil 2; HAADF and multiple elements map showing the areas from which the phases composition was extracted.

3.2 Discovery sample

3.2.1 Foil 1

The data extracted from Discovery foil 1 includes coarse gold composition and amorphous silica (aSi). The areas from where the phases composition was extracted is presented in Figure 15.

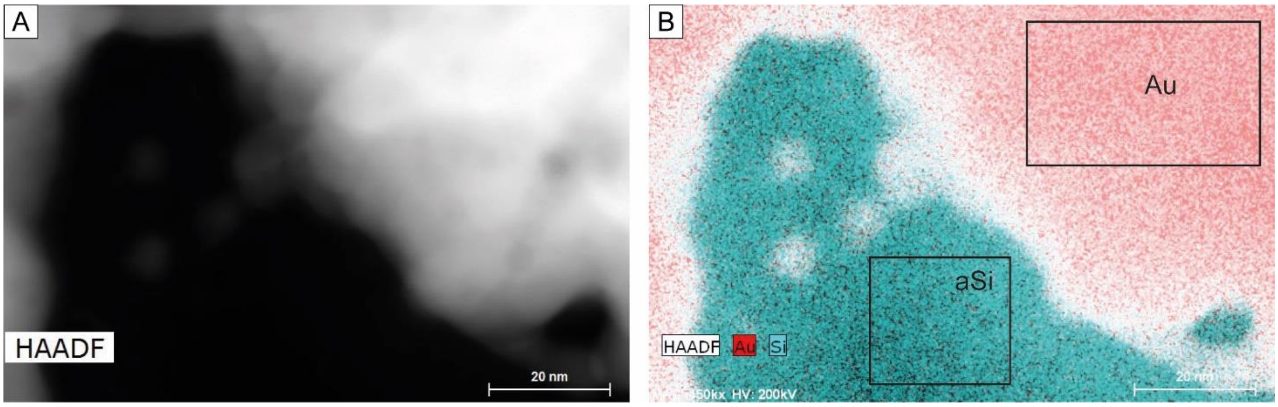

Figure 15: Discovery foil 1; HAADF and multiple elements map showing the areas from which the phases composition was extracted.

3.2.2 *Foil 2, area 1*

The data extracted from Discovery foil 2, area 1 includes coarse gold composition, amorphous silica and amorphous C. The areas from where the phases composition was extracted is presented in Figure 16. Please note that the carbon-rich area analysed is overlapping with amorphous silica and coarse gold therefore, the result cannot give a precise composition of the carbon-bearing phase. The data still shows the association of O and N with the amorphous carbonic phase.

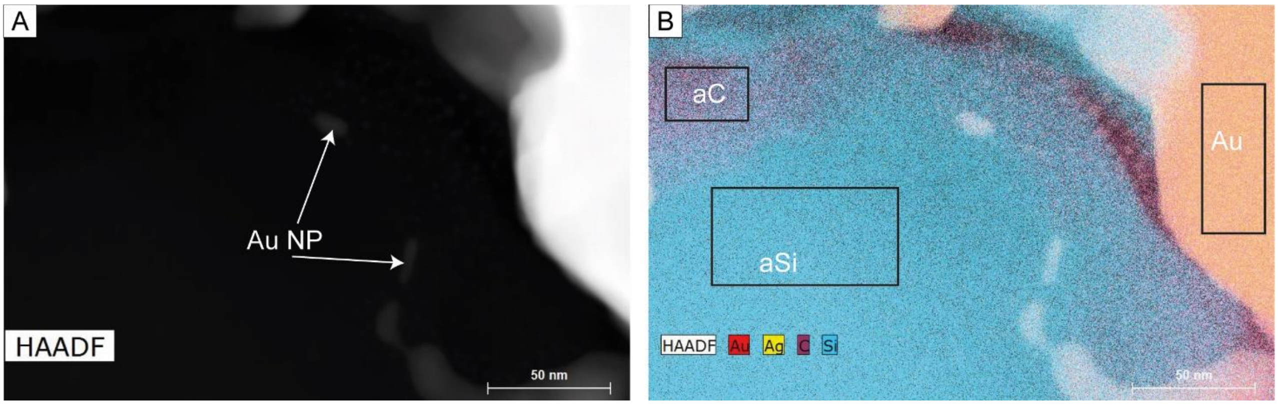

Figure 16: Discovery foil 2, area 1; HAADF and multiple elements map showing the areas from which the phases composition was extracted.

3.2.3 *Foil 2, area 2*

The data extracted from Discovery foil 2, area 2 includes coarse gold composition, Ag_2O NP and amorphous carbonic phase and amorphous carbonic phase. The areas from where the phases composition was extracted is presented in Figure 17. The area over the Ag_2O nanoparticle contains an overlap with the amorphous carbonic phase therefore, the results cannot precisely indicate the composition of the nanoparticles but indicates a higher concentration in Ag relative to Au.

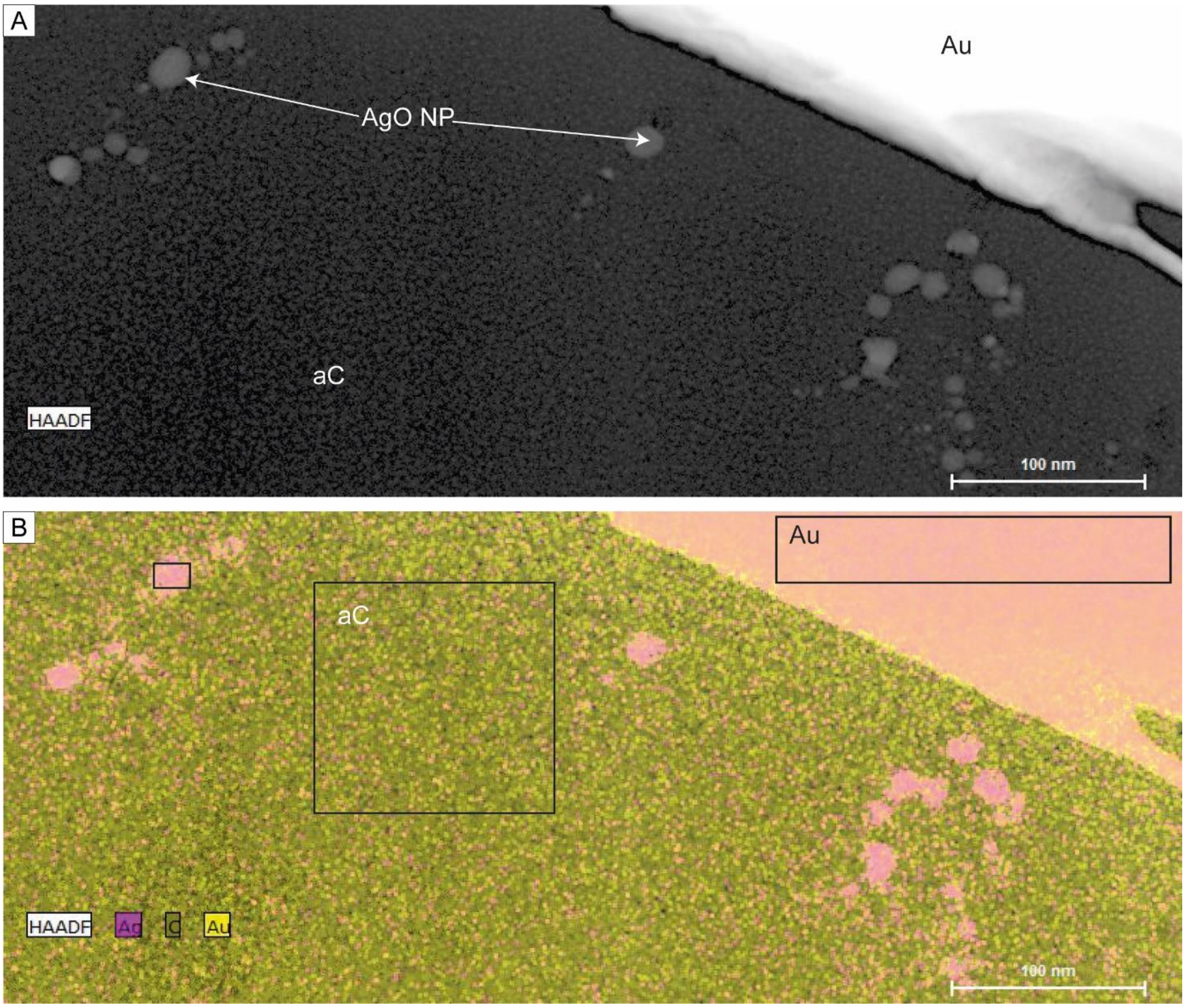

Figure 17: Discovery foil 2, area 2; HAADF and multiple elements map showing the areas from which the phases composition was extracted.

3.3 Red Lake sample

The data extracted from Red Lake foil includes coarse gold composition, Ag_2O NP and amorphous carbonic phase. The areas from where the phases composition was extracted is presented in Figure 18.

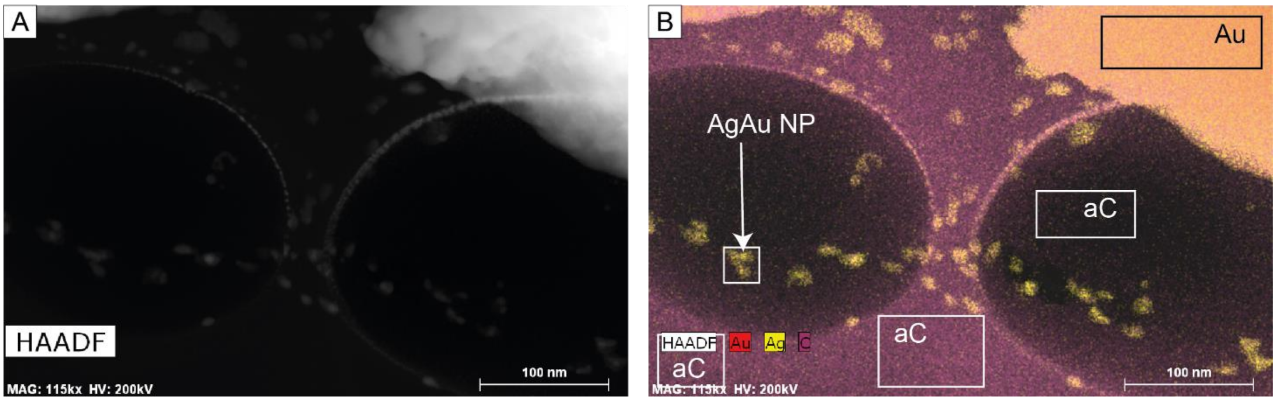

Figure 18: Red Lake foil; HAADF and multiple elements map showing the areas from which the phases composition was extracted.

3.4 Callie sample

The data extracted from Callie foil includes coarse gold composition and amorphous carbonic phase. The areas from where the phases composition was extracted is presented in Figure 19.

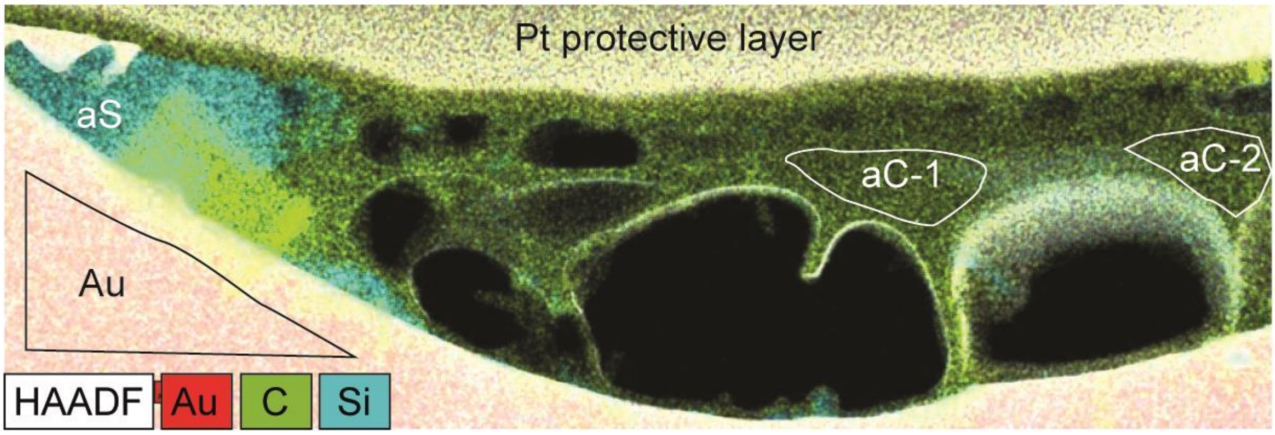

Figure 19: Callie foil; Multiple elements map showing the areas from which the phases composition was extracted.

3.5 Sixteen to One sample

The data extracted from Sixteen to One foil includes coarse gold composition and micro-crystalline carbon. The areas from where the phases composition was extracted is presented in Figure 20.

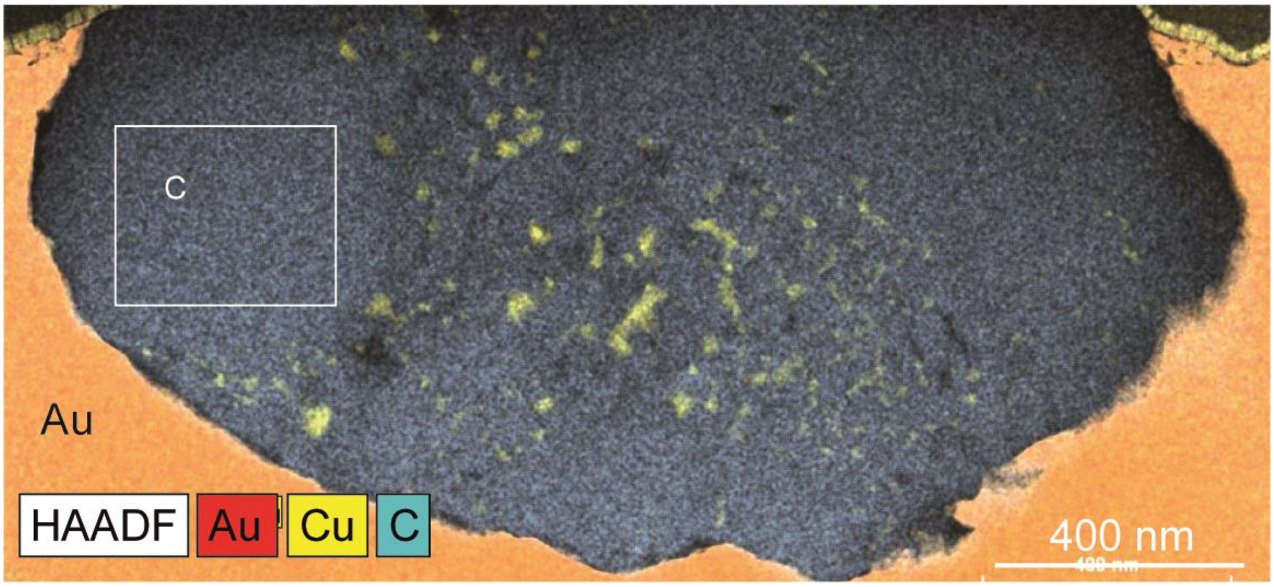

Figure 20: Sixteen to One foil; Multiple elements map showing the areas from which the phases composition was extracted.

3.6 Amorphous carbon analysis

Figure 21 summarizes the composition of amorphous carbonic phase acquired in inclusion from the Beta Hunt sample, the Callie sample, the Discovery sample and the Red Lake sample including the relative proportion of O, C and N content in At. %.

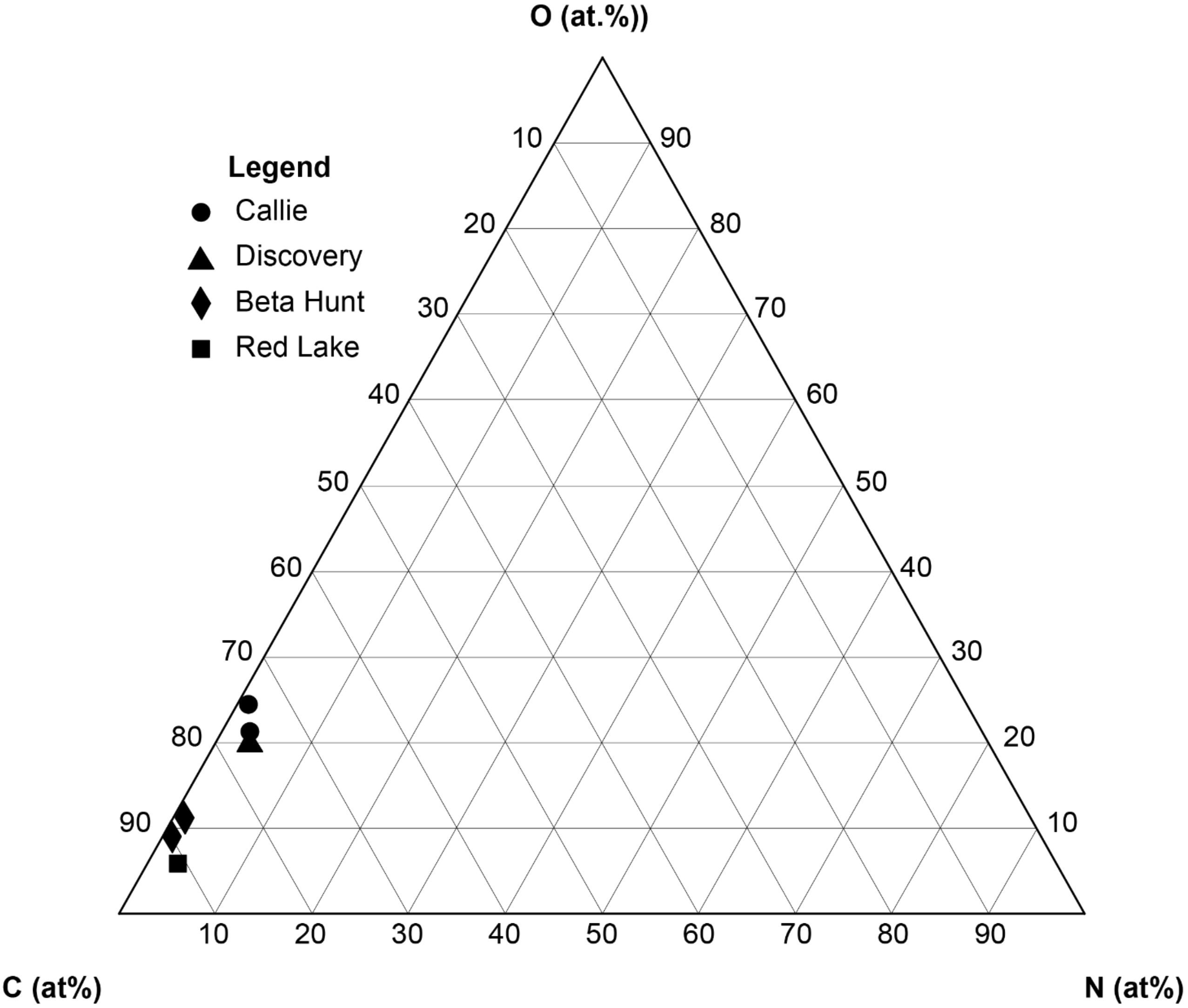

Figure 21: **Amorphous carbon composition.** Ternary diagram including O, C and N in at.% showing the composition of the amorphous carbon phase in micro-inclusions in gold.

References

1 Chi, G., Dubé, B., Williamson, K. & Williams-Jones, A. E. Formation of the Campbell-Red Lake gold deposit by H 2 O-poor, CO 2-dominated fluids. *Mineralium Deposita* **40**, 726 (2006).

2 Dubé, B. *et al.* Timing of gold mineralization at Red Lake, northwestern Ontario, Canada: New constraints from U-Pb geochronology at the Goldcorp high-grade zone, Red Lake Mine, and the Madsen Mine. *Economic Geology* **99**, 1611-1641 (2004).

3 Twomey, T. & Mcgibbon, S. The geological setting and estimation of gold grade of the high-grade zone, Red Lake mine, Goldcorp Inc. *Exploration and Mining Geology* **10**, 19-34 (2001).

4 Penczak, R. S. & Mason, R. Metamorposed Archean epithermal Au-As-Sb-Zn-(Hg) vein mineralization at the Campbell Mine, northeastern Ontario. *Economic Geology* **92**, 696-719, doi:10.2113/gsecongeo.92.6.696 (1997).

5 Chi, G., Dubé, B. & Williamson, K. *Fluid evolution and pressure regimes in the Campbell-Red Lake gold deposit, Red Lake mine trend, Red Lake, Ontario: Fluid-inclusion evidence for a protracted, highly dynamic hydrothermal system*. (Natural Resources Canada, Geological Survey of Canada, 2003).

6 Gresham, J. & Loftus-Hills, G. The geology of the Kambalda nickel field, Western Australia. *Economic Geology* **76**, 1373-1416 (1981).

7 Lucas, J. in *ABC Goldfields* (2018).

8 Witt, W. K. & Vanderhor, F. Diversity within a unified model for Archaean gold mineralization in the Yilgarn Craton of Western Australia: An overview of the late-orogenic, structurally-controlled gold deposits. *Ore Geology Reviews* **13**, 29-64, doi:https://doi.org/10.1016/S0169-1368(97)00013-9 (1998).

9 Phillips, G. & Groves, D. in *Gold'82: the geology, geochemistry and genesis of gold deposits. Symposium.* 389-416.

10 Neall, F. B. & Phillips, G. N. Fluid-wall rock interaction in an Archean hydrothermal gold deposit; a thermodynamic model for the Hunt Mine, Kambalda. *Economic Geology* **82**, 1679-1694 (1987).

11 Thébaud, N. *et al.* Protracted and polyphased gold mineralisation in the Agnew District (Yilgarn Craton, Western Australia). *Precambrian Research* **310**, 291-304, doi:10.1016/j.precamres.2018.02.013 (2018).

12 Vielreicher, N., Groves, D., McNaughton, N. & Fletcher, I. The timing of gold mineralization across the eastern Yilgarn craton using U–Pb geochronology of hydrothermal phosphate minerals. *Mineralium Deposita* **50**, 391-428, doi:10.1007/s00126-015-0589-9 (2015).

13 Bullen, W. *et al.* Economic contribution of gold mining in the Yellowknife mining district. *Geological Association of Canada Mineral Deposits Division*, 38-49 (2006).

14 Hansen, E. G. *Geochemical studies of gold mineralizing events in the Discovery-Ormsby and Clan Lake areas of the Yellowknife Greenstone Belt, Northwest Territories, Canada*, University of Missouri--Columbia, (2013).

15 Shelton, K. L., McMenamy, T. A., Hees, E. H. P. v. & Falck, H. Deciphering the Complex Fluid History of a Greenstone-Hosted Gold Deposit: Fluid Inclusion and Stable Isotope Studies of the Giant Mine, Yellowknife, Northwest Territories, Canada. *Economic Geology* **99**, 1643-1663, doi:10.2113/gsecongeo.99.8.1643 (2004).

16 Ootes, L. *et al.* The timing of Yellowknife gold mineralization: A temporal relationship with crustal anatexis? *Economic Geology* **106**, 713-720 (2011).

17 Schmeider, S., Stuart, P., Griesel, L. & Robinson, C. in *AGES 2018* (ed NT Geological Survey) (NT Geological Survey, Alice Springs, 2018).

18 Petrella, L. *et al.* Contemporaneous formation of vein-hosted and stratabound gold mineralization at the world-class Dead Bullock Soak mining camp, Australia. *Mineralium Deposita* **55**, 845-862, doi:10.1007/s00126-019-00902-7 (2020).

19 Mernagh, T. P. & Wygralak, A. S. Gold ore-forming fluids of the Tanami region, Northern Australia. *Mineralium Deposita* **42**, 145-173, doi:10.1007/s00126-006-0098-y (2007).

20 Petrella, L. *et al.* Colloidal gold transport: a key to high-grade gold mineralization? *Mineralium Deposita* **55**, 1-8 (2020).

21 Goldfarb, R. J., Groves, D. I. & Gardoll, S. Orogenic gold and geologic time: a global synthesis. *Ore Geology Reviews* **18**, 1-75, doi:http://dx.doi.org/10.1016/S0169-1368(01)00016-6 (2001).

22 Bierlein, F., Northover, H., Groves, D., Goldfarb, R. & Marsh, E. Controls on mineralisation in the Sierra Foothills gold province, central California, USA: a GIS-based reconnaissance prospectivity analysis. *Australian Journal of Earth Sciences* **55**, 61-78 (2008).

23 Ferguson, H. G. & Gannett, R. W. *Gold quartz veins of the Alleghany district, California*. (US Government Printing Office, 1932).

24 Cooke, H. R. The original Sixteen to One gold-quartz vein, Alleghany, California. *Economic Geology* **42**, 211-250 (1947).

25 Marsh, E. E. *et al.* New constraints on the timing of gold formation in the Sierra Foothills province, central California. *Ores and orogenesis: Circum-Pacific tectonics, geologic evolution, and ore deposits: Arizona Geological Society Digest* **22**, 369-388 (2008).

26 Coveney, R. M. Gold quartz veins and auriferous granite at the Oriental Mine, Alleghany District, California. *Economic Geology* **76**, 2176-2199 (1981).
